# ATF3 Sustains IL-22-Induced STAT3 Phosphorylation to Maintain Mucosal Immunity Through Inhibiting Phosphatases

**DOI:** 10.3389/fimmu.2018.02522

**Published:** 2018-11-05

**Authors:** Doaa Glal, Janaki N. Sudhakar, Hsueh-Han Lu, Ming-Che Liu, Hung-Yu Chiang, Yen-Chun Liu, Ching-Feng Cheng, Jr-Wen Shui

**Affiliations:** ^1^Institute of Biomedical Sciences, Academia Sinica, Taipei, Taiwan; ^2^Taiwan International Graduate Program (TIGP) in Molecular Medicine, National Yang-Ming University and Academia Sinica, Taipei, Taiwan; ^3^Department of Pediatrics, Taipei Tzu Chi Hospital, Buddhist Tzu Chi Medical Foundation, Taipei, Taiwan

**Keywords:** ATF3, IL-22, pSTAT3, PTPs, IBD, mucosal immunity

## Abstract

In gut epithelium, IL-22 transmits signals through STAT3 phosphorylation (pSTAT3) which provides intestinal immunity. Many components in the IL-22-pSTAT3 pathway have been identified as risk factors for inflammatory bowel disease (IBD) and some of them are considered as promising therapeutic targets. However, new perspectives are still needed to understand IL-22-pSTAT3 signaling for effective clinical interventions in IBD patients. Here, we revealed activating transcription factor 3 (ATF3), recently identified to be upregulated in patients with active IBD, as a crucial player in the epithelial IL-22-pSTAT3 signaling cascade. We found ATF3 is central to intestinal homeostasis and provides protection during colitis. Loss of ATF3 led to decreased crypt numbers, more shortened colon length, impaired ileal fucosylation at the steady state, and lethal disease activity during DSS-induced colitis which can be effectively ameliorated by rectal transplantation of wild-type colonic organoids. Epithelial stem cells and Paneth cells form a niche to orchestrate epithelial regeneration and host-microbe interactions, and IL-22-pSTAT3 signaling is a key guardian for this niche. We found ATF3 is critical for niche maintenance as ATF3 deficiency caused compromised stem cell growth and regeneration, as well as Paneth cell degeneration and loss of anti-microbial peptide (AMP)-producing granules, indicative of malfunction of Paneth/stem cell network. Mechanistically, we found IL-22 upregulates ATF3, which is required to relay IL-22 signaling leading to STAT3 phosphorylation and subsequent AMP induction. Intriguingly, ATF3 itself does not act on STAT3 directly, instead ATF3 regulates pSTAT3 by negatively targeting protein tyrosine phosphatases (PTPs) including SHP2 and PTP-Meg2. Furthermore, we identified ATF3 is also involved in IL-6-mediated STAT3 activation in T cells and loss of ATF3 leads to reduced capacity of Th17 cells to produce their signature cytokine IL-22 and IL-17A. Collectively, our results suggest that via IL-22-pSTAT3 signaling in the epithelium and IL-6-pSTAT3 signaling in Th17 cells, ATF3 mediates a cross-regulation in the barrier to maintain mucosal homeostasis and immunity.

## Introduction

Inflammatory bowel disease (IBD) is a group of chronic relapsing inflammatory conditions characterized by impaired intestinal homeostasis and abnormal stress response (1). It is believed that breach or breakdown of the epithelial barrier sets the pre-disease stage of IBD, leading to microbial translocation, immune activation and chronic inflammation at the later stages (2). Not only functioning as a physical barrier, intestinal epithelial cells (IEC) also serve as a biological barrier for maintaining homeostasis between host and microbes (3), through IEC's microbial recognition and their regulatory function in innate and adaptive immunity (4). Therefore, epithelial barrier is the major platform where cross-regulation of host-microbe interactions takes place and is believed to have a predominant role in the initiation of IBD pathogenesis (5).

The cytokine IL-22 has a unique niche in the intestine due to exclusive expression of IL-22 receptor (IL-22R) by the IEC. Stimulation of IEC by IL-22 initiates signaling transduction of IL-22R-assoicated Jak2 and Tyk2 kinases, leading to phosphorylation and activation of STAT3 (6). It is well established that IL-22-STAT3 signaling controls epithelial stem cell regeneration (7), mucosal healing during colitis (8, 9), induction of anti-microbial, cytokine or chemokine genes, and production of mucins by IEC (10). More importantly, these IL-22-mediated biological processes are associated with host defense, intestinal inflammation, metabolic disorders (obesity, diabetes, or nephropathy), tumorigenesis, and even anti-TNF therapy for IBD (10–16). Clinical relevance of IL-22 in IBD pathogenesis has been established given that some pronounced IBD risk genes, such as *Il-23, Stat3, Jak2, Tyk2, Fut2*, are involved in IL-22-STAT3 signaling, and that enhancing IL-22 pathway can improve mucosal healing and ameliorate disease progression (10). Moreover, IL-22 is considered as an ideal IBD therapeutic target because of its specific actions on epithelial cells through constitutively activating (phosphorylating) epithelial STAT3 (10). Based on these facts, therefore, a better understanding of IL-22 signaling network and its upstream or downstream transcriptional modulators will potentially improve the efficacy and safety of the current IBD drugs, or lead to identification of novel therapeutic targets for IBD treatment.

Activating transcription factor 3 (ATF3) is a stress-inducible gene which encodes a member of ATF/cyclic adenosine monophosphate (cAMP) response element (CRE)-binding (CREB) family of transcription factors (17). ATF3 has been described as a hub for the cellular adaptive-response network that has a regulatory role in disease pathogenesis (17). The fact that ATF3 is induced by lipopolysaccharide (LPS) and involved in TLR-stimulated gene transcription pinpoints its role in innate immunity, inflammation and host defense (18). Correlated to this, several studies have linked ATF3 to epithelial barrier function. In a *Drosophila* model, ATF3 was shown to control JNK and STAT signaling to maintain intestinal barrier regeneration (19). In human enterocytes, ATF3 negatively regulates Nod2-induced pro-inflammatory response (20), further supporting ATF3 contributes to barrier immunity and IBD pathogenesis (Nod2 is an IBD risk factor). Clinical correlation of ATF3 in IBD has been reported, with ATF3 expression being significantly upregulated in patients with Crohn's disease (21). While ATF3 promotes IEC apoptosis via interacting with and regulating p53 in Crohn's disease (22), ATF3 also maintains IEC survival to provide cellular defense in response to chemical stress (23). Therefore, it is still unclear how ATF3 regulates epithelial barrier function in the context of homeostasis or during intestinal stress such as inflammation. Yet, it has not been investigated if ATF3-mediated stress response in IEC involves IL-22-STAT3 signaling. Furthermore, it remains to be determined whether loss of ATF3 (i.e., loss of stress control) in gut epithelial cells could be a trigger leading to IBD pathogenesis.

In skin cancer cells, activation of STAT3 by overexpressed ATF3 enhances cell proliferation while ATF3 knockdown abolishes this effect (24). In hepatocellular carcinoma (HCC), ATF3 cooperates with SPTBN1 and SMAD3 to inhibit STAT3 activity, thereby suppresses HCC development (25). The role of ATF3 in STAT3 activation in gut epithelial cells has not been studied, nor in the context of IL-22 signaling. Intriguingly, levels of ATF3, IL-22, and IL-22R were all upregulated in IBD patients (21, 26), suggesting that ATF3 could be associated with IL-22 signaling in gut inflamed tissues. Supporting this, we first identified that IL-22 stimulation of gut epithelial cells upregulates both mRNA and protein levels of ATF3. Given that IL-22 signaling uniquely induces STAT3 phosphorylation and activation in epithelial cells, it is likely that ATF3 is involved in IL-22-mediated STAT3 activation and its downstream function, such as IEC regeneration or anti-microbial peptide production. In this study here, for the first time, we provide evidence showing that ATF3 is actively involved in the IL-22-pSTAT3 signaling cascade to maintain intestinal homeostasis at the steady state and to protect against colon tissue damage during chemical-induced colitis, by a mechanism of suppressing PTP (protein tyrosine phosphatase)-mediated STAT3 inactivation. We also identified ATF3 is acting on IL-6-mediated pSTAT3 signaling in T cells, which affects intestinal Th17 function. We concluded ATF3 orchestrates IL-22/IL-6-pSTAT3 activation in epithelium and adaptive lymphoid cells to facilitate mucosal immunity across the barrier, including barrier regeneration and production of anti-microbial peptides and Th17 cytokines. Therefore, we propose that ATF3 is a multifaceted risk factor for IBD pathogenesis.

## Materials and methods

### Reagents and antibodies

All reagents and antibodies used in this study were listed in Tables 1A,B, unless indicated elsewhere.

**Table 1:** List of the materials used in the study.

**A. ANTIBODIES**		
**Antibodies (concentration)**	**Technique**	**Source/Identifier**
Purified anti-mouse CD16/32 Antibody, clone 93, (1:500)	Flow cytometry	BioLegend (101301)
Anti-Mouse CD326 (EpCAM) Monoclonal Antibody (G8.8), eFluor 450, eBiosciense™ (1:400)	Flow cytometry	ThermoFisher Scientific (48-5791-80)
Anti-Mouse CD45 Monoclonal Antibody (30-F11), Alexa Fluor 700, eBiosciense™	Flow cytometry	ThermoFisher Scientific (56-0451-82)
Anti-Mouse CD24 Monoclonal Antibody (M1/69), APC-eFluor 780, eBiosciense™ (1:400)	Flow cytometry	ThermoFisher Scientific (47-0242-80)
Anti-Mouse Ki-67 Monoclonal Antibody (SolA15), PE-Cyanine7, eBiosciense™ (1:400)	Flow cytometry	ThermoFisher Scientific (25-5698-80)
Phospho-STAT3 (Tyr705) Monoclonal Antibody (LUVNKLA), PE, eBiosciense™	Flow cytometry	ThermoFisher Scientific/#12-9033-41
Mouse IgG2b kappa Isotype Control, PE, eBiosciense™ (clone eBMG2b) (1:100), Isotype control for pSTAT3	Flow cytometry	ThermoFisher Scientific (12-4732-81)
PE/Cy7 anti-mouse/human CD44, clone IM7	Flow cytometry	BioLegend (103029)
APC anti-mouse CD3 Antibody, clone 17A2 (1:400)	Flow cytometry	BioLegend (100235)
Anti-Human/Mouse IL-22 APC, clone IL22JOP, (1:200)	Flow cytometry	BioLegend (17-7222)
FITC anti-mouse IL-17A Antibody, clone TC11-18H10.1, (1:200)	Flow cytometry	BioLegend (506907)
CD11b Monoclonal Antibody (M1/70), PE-Cyanine7, eBioscience™ (1:400)	Flow cytometry	ThermoFisher Scientific (25-0112-81)
CD11c Monoclonal Antibody (N418), PE-Cyanine5.5, eBioscience™ (1:400)	Flow cytometry	ThermoFisher Scientific (35-0114-80)
Ly-6G/Ly-6C Monoclonal Antibody (RB6-8C5), PE-Cyanine7, eBioscience™ (1:400)	Flow cytometry	ThermoFisher Scientific (25-5931-81)
CD19 Monoclonal Antibody [eBio1D3 (1D3)], PE-Cyanine7, eBioscience™ (1:400)	Flow cytometry	ThermoFisher Scientific (25-0193-81)
CD45R (B220) Monoclonal Antibody (RA3-6B2), PE-Cyanine7, eBioscience™ (1:400)	Flow cytometry	ThermoFisher Scientific (25-0452-82)
Rat IgG2a kappa Isotype Control, PE-Cyanine7, for Ki67	Flow Cytomtery	eBioscience (25-4321-81)
Rat IgG2a kappa Isotype Control, APC, for IL22	Flow cytometry	eBioscience (17-4321-81)
FITC Rat IgG1, κ Isotype Ctrl Antibody, for IL-17A	Flow cytometry	BioLegend (400405)
Rabbit monoclonal (IgG) Ki67 (1:150)	Tissue Immunofluorescence	Abcam (ab16667)/SP6
Anti-rabbit IgG (H+L), F(ab′)2 Fragment (Alexa Fluor® 594 Conjugate) (1:300)	Tissue Immunofluorescence	Cell Signaling Technology (CST#8889)
Ulex europaeus lectin (UEA-1-FITC) (20ug/ml)	Whole mount tissue Immunofluorescence	Sigma-Aldrich (L9006)
Wheat Germ Agglutinin, Alexa Fluor® 633 Conjugate (10 ug/ml)	Whole mount tissue Immunofluorescence	ThermoFisher Scientific (W21404)
Rabbit monoclonal (IgG) ATF3 (1:1,000)	Western blot/organoid Immunofluorescence	Abcam (ab207434)/ EPR19488
Rabbit monoclonal (IgG) pSTAT3 (Y705) (1:1,000)	Western blot	Cell signaling technology (CST#9145) /D3A7
Mouse monoclonal STAT3 (1:1,000)	Western blot	Cell signaling technology (CST#9139)/124H6
Mouse monoclonal (IgG_1_) SH-PTP2 (1:500)	Western blot	Santa Cruz (sc-7384)/B-1
Mouse monoclonal (IgG_1_) PTP-MEG2 (1:500)	Western blot	Santa Cruz (sc-271052)/D-5
Mouse monoclonal (IgG_2a_) PAC-1 (1:500)	Western blot	Santa Cruz (sc-32776)/ 4O21
Mouse monoclonal β-actin (1:1000)	Western blot	Santa Cruz (SC-47778)/C4
Rabbit monoclonal Histone H3 (1:2,000)	Western blot	Cell signaling technology (CST#4499)/D1H2
Rabbit monoclonal GAPDH (1:1,000)	Western blot	Cell signaling technology (CST#5174)/D16H11
**B. REAGENTS**		
**Reagents/Resources:** ***Organoid***	**Technique**	**Source/Identifier**
Corning® Matrigel® Growth Factor Reduced (GFR) Basement Membrane Matrix, Phenol Red-free (10ml)	Organoid culture	Becton Dickinson (#356231)/Lot.5033306
Jagged−1 (188–204), Notch Ligand	Organoid culture	AnaSpec (AS-61298)
Gibco™ Advanced DMEM/F-12	Organoid culture media	ThermoFisher Scientific/12634010
GlutaMAX™ Supplement	Organoid culture media	Life Technologies (35050-061)
N-Acetyl-L-cysteine	Organoid culture media	Sigma Aldrich (A9165)
B-27	Organoid culture media	Life Technologies (17504-044)
N-2	Organoid culture media	Life Technologies (17502-048)
Bovine Albumin Fraction V Solution (7.5%)	Organoid culture media	Life Technologies (15260-037)
ROCK Inhibitor (Y-27632)	Organoid culture media	Sigma-Aldrich (Y0503)/Lot. 6123002
EGF Recombinant Mouse Protein	Organoid culture media	Life Technologies (PMG8041)/Lot. 5264009
Recombinant Mouse HGF Protein	Organoid culture media	R&D system (2207-HG-025)/Lot. 5313012
TGF-b inhibitor (A 83-01)	Organoid culture media	R&D system (2939)/Lot. 6214005
Recombinant Murine Wnt-3a	Organoid culture media	PeproTech (315-20)
Recombinant Murine Noggin	Organoid culture media	PeproTech (250-38)
Recombinant Mouse R-Spondin 1 Protein, CF	Organoid culture media	R&D systems (3474-RS-050)
HEPES (1 M)	Organoid culture media	Life Technologies (15630-080)
TrypLE™ Select (1X), no Phenol Red	Organoid culture	Life Technologies (12563-011)
Gentle Cell Dissociation Reagent	Organoid transfer/ICC staining	StemCell (#07174)
AERRANE (isoflurane, USP)	Organoid transfer /Inhalation anesthesia	Baxter Healthcare of Puerto Rico (#N029E423)
**Reagents/Resources:** ***Kits***	**Technique**	**Source/Identifier**
*in situ* Cell Death Detection Kit, TMR red kit	TUNEL assay	Sigma-Aldrich/ 000000012156792910
iSript TM cDNA synthesis kit	Real Time PCR	Bio-Rad (1708890)
T-Pro Bradford Protein Assay kit	Western blot	OmicsBio (JB04-D002)
T-Pro LumiLong Plus Chemiluminescence Detection kit	Western blot	OmicsBio (JT96-K004M)
Pierce™ Biotin 3′ End DNA Labeling Kit	EMSA	ThermoFisher Scientific (89818)
LightShift™ Chemiluminescent EMSA Kit	EMSA	ThermoFisher Scientific(20148)
**Reagents/Resources:** ***Common***	**Technique**	**Source/Identifier**
eBiosciense™ IC Fixation Buffer	Flow cytometry	ThermoFisher Scientific (00-8222-49)
Mouse IL-22 Recombinant Protein, eBioscience™	Cell stimulation	ThermoFisher Scientific (14-8221-63)
Recombinant Mouse IL-6 (carrier-free)	Cell stimulation	BioLegend (575702)
Gibco™ RPMI 1640 Medium, Powder	LPL isolation/culture media	ThermoFisher Scientific (31800022)
Mouse IL-23 Recombinant Protein, eBioscience™	LPL stimulation	ThermoFisher Scientific (14-8231-63)
PHORBOL 12-MYRISTATE 13-ACETATE (PMA)	LPL stimulation	Sigma-Aldrich (P8139)
Ionomycin from Streptomyces conglobatus	LPL stimulation	Sigma-Aldrich (I9657)
Brefeldin A (BFA)	LPL culture	Sigma-Aldrich (B6542)
BD Cytofix/Cytoperm™	LPL flow cytometry intracellular staining	BD Biosciences (554722)
BD Perm/Wash™	LPL flow cytometry intracellular staining	BD Biosciences (554723)
Dulbecco's Modified Eagle's Medium—low glucose	Cell culture media	Sigma-Aldrich (D6046)
Fetal bovine serum (FBS)	Cell Organoid culture media	Life Technologies (10437-028)
PolyJet™ *in vitro* DNA Transfection Reagent	CRISPR	SigmaGen (SL100688)
Immobilon-P PVDF Membrane	Western blot	MERCK (IPVH00010)
Biodyne™ B Nylon Membrane, 0.45 μm, 8 × 12 cm	EMSA	ThermoFisher Scientific (77016)
ProLong® Diamond Antifade Mountant	Immunofluorescence	Molecular Probes (P36961)
MP Premium Dextran Sulfate Sodium Salt (DSS, 100 g/bottle)	*in vivo*/*in vitro* experiments	MP Biomedicals (0216011080)
LiberaseTM	LPL isolation	Roche (05401127001)
Deoxyribonuclease I (DNase I)	IECs/LPL isolation	Sigma-Aldrich (SI-DN25-1G)
TRIzol RNA Isolation Reagent	RNA extraction	ThermoFisher Scientific (15596018)
SYBR Green PCR Master Mix (5 ml × 10)	Real time PCR	ABI (4368708)
2x RIPA buffer I (pH = 7.4) 2x concentrate	Western blot	OmicsBio (RB4475)
Halt™ Protease and Phosphatase Inhibitor Cocktail, EDTA-free (100X)	Western blot/EMSA	ThermoFisher Scientific (78441)
**Mouse Primers**	**Technique**	**Sequence**
**C.MOUSE PRIMERS SEQUENCE**		
**Mouse Primers**	**Technique**	**Sequence**
ATF3	CRISPR	Forward: 5′CACCgCC ATCGGATGTCCTCTGCGC 3′
		Reverse: 5′AAACGCGCAGAGGACATCCGATGGc 3′
ATF3	Real time PCR	Forward: TTACCGTCA ACA ACA GAC CC
		Reverse: TCA GCT CAGCATTCACAC TC
Reg3g	Real time PCR	Forward: TCA GGT GCA AGG TGA AGT TG
		Reverse: GGCCACTGTTACCACTGC TT
S100A8	Real time PCR	Forward: TGT CCT CAG TTT GTG CAG AAT ATA AA
		Reverse: TCA CCA TCG CAA GGA ACT CC
S100A9	Real time PCR	Forward: GGT GGA AGC ACA GTT GGC A
		Reverse: TCC AGG TCCTCCATGATG
β-defensin 3	Real time PCR	Forward: GTC TCC ACC TGCAGC TTT TAG
		Reverse: AGG AAA GGA ACT CCA CAA CTG C
GRP78	Real time PCR	Forward: ACTTGGGGACCACCTATTCCT
		Reverse: ATCGCCAATCAGACGCTCC
sXBP1	Real time PCR	Forward: CTGAGTCCGAATCAGGTGCAG
		Reverse: GTCCATGGGAAGATGTTCTGG
CHOP	Real time PCR	Forward: CCACCACACCTGAAAGCAGAA
		Reverse: AGGTGAAAGGCAGGGACTCA
Cyclin D	Real time PCR	Forward: GCAAGCATGCACAGACCTT
		Reverse: GTTGTGCGGTAGCAGGAGA
C-Myc	Real time PCR	Forward: TAGTGCTGCATGAGGAGACA
		Reverse: GGTTTGCCTCTTCTCCACAG
TCF7	Real time PCR	Forward: ATCCTTGATGCTGGGATTCTG
		Reverse: CTTCTCTTGCCTTGGGTTCTG
Sox9	Real time PCR	Forward: CTGGAGGCTGCTGAACGAGAG
		Reverse: CGGCGGACCCTGAGATTGC
Fut2	Real time PCR	Forward: TGC ACT GGCCAG GAT GAA
		Reverse: GCGCTA GAG CGT TGT GCA T
IL-22	Real time PCR	Forward: TCG CCT TGA TCTCTCCAC TC
		Reverse: GCT CAGCTC CTG TCACAT CA
IL-22R1	Real time PCR	Forward: CTACGTGTGCCGAGTGAAGA
		Reverse: AAGCGTAGGGGTTGAAAGGT
IL-10R2	Real time PCR	Forward: GCCAGCTCTAGGAATGATTC
		Reverse: AATGTTCTTCAAGGTCCAC
IL-6	Real time PCR	Forward: ACA AGT CGG AGG CTT AAT TAC ACA T
		Reverse: TTG CCA TTG CAC AAC TCT TTT C
IL-6R1	Real time PCR	Forward: AAGCAGCAGGCAATGTTACC
		Reverse: CATAAATAGTCCCCAGTGTCG
gp130	Real time PCR	Forward: ATAGTCGTGCCTGTGTGCTTA
		Reverse: GGTGACCACTGGGCAATATG
IL-17A	Real time PCR	Forward: TCC AGA AGG CCC TCA GAC TA
		Reverse: TTC ATT GCG GTG GAG AGT C
L32	Real time PCR	Forward: GAA ACT GGC GGA AAC CCA
		Reverse: GGA TCTGGC CCT TGA ACC TT
GAPDH	Real time PCR	Forward: GTA TGA CTCCACTCA CGG CAA ATT

### Mice

Global ATF3 knockout mice were kindly provided by Dr. Tsonwin Hai (Ohio State University, Columbus, United States) and obtained from Dr. Ching-Feng Cheng (Tzu Chi University, Taiwan). ATF3 knockout mice were backcrossed to C57BL/6J (B6) mice for at least seven generations before our study (27). ATF3^flox/flox^ mice were also kindly provided by Dr. Tsonwin Hai and were backcrossed to C57BL/6J (B6) mice for six generations before our study (28). All mice were at the age of 2~3 months old when analyzed and were maintained under standard conditions at Academia Sinica, Animal care and all experimental protocols were approved by the Institutional Animal Care and Use Committee (IACUC) at the Institute of Biomedical Sciences, Academia Sinica, Taiwan.

### DSS induced colitis

For survival studies, mice were given 2% dextran sodium sulfate (DSS) (MP Biologicals, molecular mass 36-40 kDa) in drinking water for two cycles over 20 days as follows: day-0 to day-5 (cycle 1) of 2% DSS followed by 5 days of normal drinking water, then switch back to 2% DSS (cycle 2, 5 days) then switched to normal drinking water (5 days). Disease activity index (DAI), slightly modified from previously described protocols (29, 30), was measured daily after the start of DSS treatment as a sum score of (a) weight loss percentage [scale: 0–4, no loss (0), 1–5% (1), 5–10% (2), 10–20% (3), >20% (4)], (b) stool consistency [scale: 0-4, hard (0), soft (2), very soft (3), diarrhea (4)], and (c) rectal blooding [scale: 0-4, no blood in feces (0), trace blood in feces (2), dark red colored feces (3), gross bleeding (4)].

Colonoscopy image: At day-8 of DSS intake, mice were anesthetized by isoflurane inhalation (one mouse at one time), then the mouse was moved to a chamber with cotton soaked with isoflurane for anesthesia maintenance while performing colonoscopy. Images were recorded as videos by using endoscopy system (TESALA AVS, Olympus, Tokyo, Japan) attached to an air pump to achieve regulated inflation of the colon, and endoscope was coated with a sterile thin layer of Vaseline as a lubricant to avoid mucosal irritation.

H&E staining: At day-8 of DSS intake, mice were sacrificed, colons were washed and cleaned with gentle rinsing by 1x PBS to remove all the feces, colon fragments (~0.5 cm long) were fixed with 4% paraformaldehyde, and sent to the Pathology Core Lab at the Institute of Biomedical Sciences, Academia Sinica, Taiwan for H&E sections preparation. Histological scores were calculated as previously described (30), with some modifications as follows: inflammation severity (scale: 0–4), inflammation extent (scale: 0–4) (none, mucosa, submucosa, transmural), crypt loss (scale: 0–4) (0, 1/3, 2/3, surface epithelium present, damage of surface epithelium), percent of are affected (scale: 0–4) (0, 25, 50, 75, and 100%).

### Isolation of intestinal epithelial cells (IEC) and flow cytometry

IECs were isolated using a modified version of a previously described protocol (31). Intestine tissue was flushed by 1x PBS, cut open into small fragments (~1 cm) which were washed in 50 ml falcon tube by hand shaking few times in 1x PBS. The tissues were transferred into a new pre-cold 50 ml falcon tube containing 30 mM EDTA/1x PBS and 1.5 mM DTT then incubated on ice for 20 min. Then the supernatants were discarded and the tissues were transferred to a new pre-warmed 50 ml falcon tube containing 30 mM EDTA/1x PBS and vortex for 30 s, the tissues were then incubated at 37°C for 10 min and vortex briefly 5 times to isolate epithelial cells. Supernatants were collected to a new 50 ml falcon tube and pelleted by centrifugation. The cells were then digested into single cells by incubating in 1x HBSS containing 0.3 U/ml Dispase at 37°C for 10 min. After 10 min, to stop digestion reaction, 100 ug DNase I and 5% FBS were added and the cells were pelleted, and diluted in 1 ml staining buffer (1x PBS with 2% FBS). 0.5 ml was used for quantitative PCR (QPCR) analysis, and the rest of the cells were used for flow cytometry analysis as follows: samples were incubated for 8 min with blocking antibody (anti-CD16/32) in staining buffer on ice, followed by washing in 1 ml staining buffer, centrifuged at high speed (12,000 × g) for 10 s, the pellets were then re-suspended in staining buffer containing surface antibody cocktail with the following fluorescent antibodies: EpCAM-eF450, CD45-AF700, CD24-APC-Cy7 for 20 min on ice. Next, cells were washed with 1 ml staining buffer and fixed in 4% PFA for 15 min before intracellular staining. The fixed single cells were incubated in 1x PBS containing 0.1% saponin and anti-Ki67 antibody for 30 min. Data were acquired using a LSRII flow cytometer (BD Biosciences) and analyzed with FlowJo software.

### Immunofluorescence staining of tissue sections

Mouse tissues were prepared as described above in the DSS-colitis section. Paraffin sections were first de-paraffinized by three times wash in xylene (5 min each), followed by two times wash of 100% ethanol (10 min each), then two times wash in 95% ethanol (10 min each), and then washed three times in ddH2O. Then the sections were dewaxed and heat-mediated antigen retrieval was performed by incubation in 10 mM sodium citrate buffer pH 6.0 for 15 min at a sub-boiling temperature. Slides were then allowed to cool down for 10 min at RT and then washed three times in ddH2O before being blocked with blocking buffer (1x PBS/5% normal goat serum) for 1 h, followed by permeabilization in 1x PBS/0.3% Triton X-100. After 15 min, the sections were incubated with primary rabbit anti-Ki67 (Abcam) in dilution buffer (1x PBS/1% BSA/0.3% Triton X-100) at 4°C overnight. Next day, the sections were washed three times with 1x PBS (5-10 min each) followed by incubating with fluorophore-conjugated with anti-rabbit Alexa Fluor-594 at room temperature for 2 h. To visualize the nucleus, slides were washed as before and counterstained with Hoechst 33342 (1 ug/mL in PBS) for 10 min and cover slips were mounted on the slides by using ProLong Gold Antifade Mountant (Invitrogen). Confocal images were obtained with a Carl Zeiss LSM 700 stage imaging system under a 20, 40, or 100x oil-immersion objectives.

### Tunel assay

Mouse tissues were prepared as mentioned above in the DSS-colitis section. Terminal deoxynucleotidyl transferase (TdT) dUTP Nick-End Labeling (TUNEL) assay was performed according to instructions in *in situ* Cell Death Detection, TMR red kit (Roche). Paraffin-Embedded tissues sections were processed as mentioned above in the section of Immunofluorescence staining. After tissue permeabilization, the sections were incubated with TUNEL reaction mixture at dark in humidified atmosphere, 37°C for 1 h. To visualize the nucleus, slides were washed as before and counterstained with Hoechst 33342 (1 ug/mL in PBS) for 10 min and cover slips were mounted on the slides by using ProLong Gold Antifade Mountant (Invitrogen). Confocal images were obtained with a Carl Zeiss LSM 700 stage imaging system.

### Whole mount tissue

Small sections of the terminal ileum (0.5–1 cm long) were cut, fixed immediately with 4% PFA when processing multiple sections at the same time, each section was then washed with 1x PBS and cleaned with feces and fat tissues removed. Each segment was cut open, washed again to remove the mucus, placed on tissue paper so that the luminal side faces upward and incubated overnight in 4% PFA at 4°C. Next day, tissues were washed in 1x PBS 3 times for 10 min with shaking at 60 rpm. A solution of fluorescence-conjugated UEA-1 (20 μg/mL) and WGA (10 μg/mL) in antibody dilution buffer was prepared. Each segment was mixed with 1 ml of antibody mixture and incubated on ice for 3 h for each antibody. After washing in 1x PBS, segments were placed on glass slides, with the apical luminal surface facing upward, residual PBS was removed gently by Kim wipes, then ProLong Gold Antifade Mountant (Invitrogen) was added and the tissue segments were sandwiched by placing cover slip over them. Confocal images were obtained with a Carl Zeiss LSM 700 stage imaging system.

### Intestinal crypt isolation and organoid culture

Intestinal crypt isolation was based on the protocol by Sato et al with some modifications (32). Mice were euthanized with CO_2_ and the abdomen was cut open to separate ~12 cm of distal small intestine (whole Ileum) and ~5 cm of distal large intestine (distal colon). Peyer's patches and fat tissues were removed and organs were cut opened longitudinally and washed with PBS. The colon was scratched with a cover slip a few times to remove the upper layer of epithelial cells. The intestines were then cut into 1 cm pieces and placed into 50 ml falcon tubes and washed vigorously by shaking in Hank's Balanced Salt Solution (HBSS) until the supernatant was clear. The tissues were then incubated in 30 mM EDTA/1x PBS with shaking at 150 rpm for 5 min in 37°C. The tissues were then transferred into a new 50 ml falcon tube containing cold 1x PBS and put horizontally under ice with shaking at 150 rpm for 15 min. The supernatant was then replaced with 10 ml wash buffer (1x PBS at pH 7.4, 1x penicillin/ streptomycin, 50 ug/ml Gentamicin, 0.1% BSA), vortexed for 6 times and then the supernatant was transferred to a new 50 ml falcon tube on ice. This process was repeated 3–5 times. For ileum only, crypts were filtered through a 70 μm strainer to remove villi. After that, the collected supernatant was centrifuged at 50 g for 5 min at 4°C and the pellet was resuspended gently in 5–10 ml wash buffer. For colon only, colon crypts were mildly digested by incubating in 1x TrypLE Select Enzyme (Gibco) for 1 min at room temperature into small cluster of cells. The digestion was stopped by mixing the crypts with 10 ml wash buffer and pelleted by centrifuge at 300 g for 5 min at 4°C. The total crypt number was measured by plating 100 ul crypt suspension on petri dish and counted under a microscope. An aliquot of cell suspension containing approximately 500 crypts was centrifuged at 300 g for 5 min at 4°C and pellets were gently mixed with Matrigel (BD Biosciences) containing Jagged-1 peptide (50 ul Matrigel used for 500 crypts). The mixture was then plated in 24-well plate and incubated in 37°C for 10 min before adding organoid growth medium. Organoid growth medium was prepared (1:1 mixture of basal culture medium and WNR conditioned medium) and added to each well. Basal culture medium: (advanced DMEM/F-12 medium supplemented with 100 U/ml penicillin, 100 ug/ml streptomycin, 50 ug/ml Gentamicin, 2 mM GlutaMAX, 10 mM HEPES, 1 mM N-acetyl-L-cysteine, 1x B-27 Supplement, 1x N-2 Supplement, and 1% BSA). WNR conditioned medium: [WNT, R-spondin, Noggin medium, derived from stable cell lines, supplemented with 10 uM Y-27632 (ROCK inhibitor), 50 ng/ml mEGF, 0.5 uM A-83-01 (for ileum crypts), or 100 ng/ml murine Hepatocyte Growth Factor (mHGF) (for colon crypts)]. Medium was replaced every 2 days.

### Organoid transplantation

The procedure of colon organoid transplantation was based on the protocol described by Yui et al. with some modifications (33). Crypts were isolated from distal colon tissues of 8–12 weeks old mice as described above and organoids were cultured for 6 days and used as donor organoids. The recipient ATF3-deficient mice were fed with 5 days of 1.5% DSS water followed by 9 days of regular water. At day-6 and day-8 post DSS intake, Matrigel containing organoids were dissolved in cell recovery solution (Stem Cell Technologies) by pipetting and cell suspension was transferred to a 50 ml falcon tube, followed by shaking at 100 rpm on ice for 1 h and then centrifuged at 300 g for 5 min at 4°C. The pellet was washed twice in 10 ml of cold 1% BSA/1x PBS by spinning at 20 g for 5 min at 4°C. The pellets was then resuspended in 180 ul of cold PBS and transferred to an eppendorf, followed by adding 20 ul of Matrigel, 1 ul of Jagged-1 peptide, and 1 ul of Y-27632 inhibitor. The collected organoids (~1,000 cells) were transferred to colon of the recipient mice (~5 cm deep) through intra-rectal injection by using 1 ml syringe and a thin flexible catheter (35 cm in length and 1.2 mm in diameter, Terumo Inc.) at day-6 and day-8 post DSS administration. After infusion, the anal verge of recipient mouse was glued with Vetbond Tissue Adhesive (3 M Vetbond) for 6 h to prevent the injected organoids from being excreted from the colon. The survived recipient mice were sacrificed at day-14 for analysis.

### Intracellular organoid staining of pSTAT3

Intracellular staining of STAT3 phosphorylation in organoids was performed as previously described (7). In brief, organoids were mechanically disrupted into crypt suspension in cell dissociation buffer (Stem Cell Technologies), by pipetting cell suspension in 50 ml falcon tube and rotating on ice at 100 rpm for 1 h, followed by centrifuging at 300 g for 5 min at 4°C. The pellet was then stimulated with 200 ul of organoid culture media containing rmIL-22 (20 ng/ml, eBioscience) for 20 min. After stimulation, the cells were fixed by adding an equal volume of intracellular fixation buffer (eBiosciense) and incubating in dark at RT for 1 h. Single cell suspension was made by rotating the cells in TrypLE (Gibco) at 100 rpm for 15 min at 37°C and then passed through a 70 μm strainer. Permeabilization was performed by adding 1 ml of ice-cold methanol for 30 min at 4°C. Cells were thoroughly washed with PBS before staining with a blocking antibody (CD16/32) followed by intracellular staining with PE-pSTAT3 and PE-IgG2b isotype control antibodies (eBioscience). Samples were immediately analyzed by LSR-II flow cytometer.

### Generation of ATF3-deficient CMT-93 cells by crispr-Cas9

CMT-93 cells, a murine rectal-carcinoma cell line obtained from ATCC, were maintained in Dulbecco's Modified Eagle's Medium (DMEM) supplemented with 10% fetal bovine serum (FBS), 100 U/mL of penicillin, 100 ug/ml of streptomycin and 2 mM GlutaMAX (all reagents obtained from Gibco). Cells were passaged (1:10) after reaching 70~80% confluence. sgRNA design and cloning were performed based on a protocol by Ran et al. (34), with a CRISPR design tool (http://crispr.mit.edu). The sequences of oligos were: Forward primer: 5′-CACCgCCATCGGATGTCCTCTGCGC-3′, Reverser primer: 5′AAACGCGCAGAGGACATCCGATGGC-3′. ATF3 targeting sequences were cloned into BbsI-digested pSpCas9(BB)-2A-GFP plasmid (Addgene, px458), which was a gift from Dr. Liuh-Yow Chen at Academia Sinica, Taiwan. The px458-based constructs were introduced into CMT-93 cells by PolyJet^TM^ *in vitro* DNA Transfection Reagent using advanced protocol (SignaGen). Single GFP^+^ cells were sorted into 96-well plate to obtain single cell clones by FACSAria cell sorter (BD Biosciences) at IBMS core facility, Academia Sinica, Taiwan. The ATF3-deficient cells were confirmed by Western blot analysis.

### Silencing the mouse PTPN11 (SHP2) gene expression in CMT93 cells

The lentiviral RNAi system was provided by the National RNAi Core Facility, Academia Sinica, Taipei, Taiwan. To silence the Ptpn11 (NM_011202) gene expression, lentiviral vector pLKO_TRC005 encoding small hairpin RNA (shRNA) specific to Ptpn11 (Clone ID: TRCN0000327986) was used. The traget sequence for Ptpn11 was 5′-CGTGTTAGGAACGTCAAAGAA-3′ (nts 1,332–1,352), and the double-stranded oligonucleotide containing the following shRNA sequence 5′-CCGGCGTGTTAGGAACGTCAAAGAACTCGAGTTCTTT GACGTTCCTAACACGTTTTTG-3′ was introduced into the lentiviral vector. To produce recombinant lentiviruses, HEK293T cells were co-transfected with lentiviral vector carrying gene-specific shRNA (2.5 ug), Gag and Polymerase (RT) expression plasmid pCMV-ΔR8.91 (2.25 ug) and VSV-G envelope glycoprotein expression plasmid pMD.G (0.25 ug) by using PolyJet^TM^ *in vitro* DNA Transfection Reagent (SignaGen Laboratories #SL100688). After 72 h the medium was harvested, aliquoted and stored at −80°C. To generate Ptpn11 specific gene knockdown cells, CMT-93 ATF-3 KO cells were grown to 80% confluency and were infected with recombinant lentiviruses encoding Ptpn11 specific siRNAs with an MOI >6 in the presence of 8 ug/mL polybrene (Sigma #H9268) for 24 h. The cells were rinsed in DMEM and allowed to grow for another 36 h in the growth medium. Subsequently, the cells were selected in the growth medium containing 2 ug/mL of puromycin dihydrochloride (Gibco^TM^ #A11138) for 1 week. The puromycin-resistant cells were collected and gene knockdown in cells was determined by immunoblotting with anti-Ptpn11 antibody (Santa Cruz #sc-7384).

### Wound healing assay

Approximately 1.2 × 10^6^ of wild-type or ATF3^−/−^ CMT-93 cells were seeded in triplicate into a 6-well plate and grown for 12 h in complete DMEM. Cell monolayer was scraped to form a wide strip (~1,100 μm) by using a standard 1,000 ul pipette tip. The wounded monolayers were washed to remove non-adherent cells. Each wound was photographed and measured at two pre-marked positions per well at time point of 0 h and time point of 24 h post wounding. Wound recovery was expressed as percentage of wound closure after 24 h relative to 0 h post scratch. Each dot shown in the figure represents one wound in each well and the data is representative of at least two independent experiments.

### Cell proliferation assay

Approximately 0.2 × 10^6^ of wild-type or ATF3^−/−^ CMT-93 cells were seeded, serum starved for 24 h and this was considered as day-0. Serum-free medium was replaced with complete DMEM containing 10% FBS after starvation and the cells were grown for another 24 h in complete DMEM with or without 5% DSS (day-1). DSS-treated cells were washed twice with PBS and allowed to recover in complete DMEM for another 24 h (day-2), whereas untreated cells were simply grown in complete DMEM until day-2. Total cell proliferation was calculated as percentage relative to the number of cells seeded. Cell number was determined by using 0.2% tryphan blue staining in cells and counted by Cellometer (Nexcelom Bioscience).

### Isolation, culture, and stimulation of lamina propria cells

Colon lamina propria (LP) cells were isolated based on a protocol by Moro et al. (35). In brief, dissected colons were washed with ice-cold 2% FBS/1x PBS wash buffer, cut into small pieces, and transferred to a 100 ml glass bottle containing 40 ml of depletion buffer (1 mM EDTA/1x PBS). Colon tissues were put for stirring at 37°C for 30 min, then washed in 1x PBS by hand shaking in a 50 ml falcon tube to ensure removal of epithelial cells. Next, colon pieces were minced by a scissor in eppendorf with 1 ml of digestion buffer, RPMI medium with 2% FBS, Liberase^TM^ (15 ug/ml, Roche), DNase1 (50 ug/ml, Sigma), then transferred to a 50 ml flask with 9 ml of digestion buffer and put for stirring at 37°C for 30 min. The supernatant was filtered through a 70 μm strainer into a new 50 ml falcon tube on ice and the tissues were homogenized through an 18G needle then again stirred in 10 ml of digestion buffer for another 30 min. The supernatant was centrifuged at 600 g for 6 min at 4°C and the pelleted LP cells were re-suspend in complete RPMI for cell counting, the cells were then divided into to 3 portions for RNA extraction, unstimulated or stimulated culture in V-bottom 96-well plate for flow cytometry analysis of cytokine production. After 3 h stimulation with IL23/PMA/Ionomycin cocktail, the cells with briefly re-suspended by multi-channel pipette before spinning down at 1,500 rpm for 5 min at 4°C and washed once with staining buffer. 50 ul staining buffer with blocking antibody (CD16/32) was added to the cells for 10 min before performing surface staining (EpCAM/CD45/Lineage markers) for 20 min in dark at 4°C. For intracellular staining, the cells were fixed in BD CytoFix/CytoPerm buffer for 30 min at 4°C, washed once with 200ul of BD Perm Wash Buffer followed by spinning down, 1,800 rpm, 6 min for intracellular staining mixture (APC-IL22/FITC-IL17A) using 1x Wash Buffer. After washing in staining buffer, the cells were re-suspended in 150 ul staining buffer and immediately analyzed by LSR-II flow cytometer.

### RNA extraction and quantitative real-time PCR analysis

Total RNA was isolated using TRIzol reagent (Invitrogen) according to the manufacturer's instructions. One microgram of RNA was reverse-transcribed to cDNA with iScript^TM^ cDNA synthesis kit (Bio-Rad). Transcript levels of the target genes were analyzed using Power SYBR® Green PCR Master Mix (Applied Biosystems) on a StepOnePlus Real-Time PCR System (Life Technologies). Primer sequences were listed in Table 1C.

### Western blot analysis

Cells were cultured in complete DMEM till 70–80% confluence and serum starved for 5 h before stimulation. Medium was replaced with complete medium with or without recombinant mouse IL-22 (50 ng/ml, eBioscience) for 10 min stimulation. Cells were then washed with 1x PBS and immediately scraped from the plate into 1x RIPA lysis buffer containing protease/ phosphatase inhibitors (Thermo Fisher). The lysates were then sonicated and centrifuged at 14,000 g for 15 min and the supernatants were collected and quantified with a Bio-rad protein assay kit for Western blot analysis. Protein samples were separated on SDS-PAGE gels and transferred to polyvinylidene fluoride membrane. Membranes were blocked with 5% skim dried milk in Tris buffered saline buffer (TBST: 50 mM Tris-HCl, pH 7.4, 150 mM NaCl, 0.1% Tween 20) and incubated overnight with the following primary antibodies: Anti-ATF3 (Abcam ab207434), pSTAT3 (CST#9145), STAT3 (CST#9139), PTP-SHP-2 (Santa Cruz #sc-7384), PTP-MEG2 (#sc-2271052), PAC-1 (#sc-32776), β-actin (#sc-47778), Histone H3 (CST#4499), GAPDH (CST#5174), α-tubulin (#abcam2791, Clone DM1A). After washing with TBST, blots were incubated with HRP conjugated secondary antibodies. Detection was achieved using a chemiluminescence substrate (Omics Bio) and images were acquired using ImageQuant™ LAS 4,000 camera.

### Electrophoretic mobility shift assay (EMSA)

Wild-type and ATF3^−/−^ CMT93 cells were cultured till 80% confluence and serum starved for 5 h before IL-22 (50 ng/ml) stimulation. Cytoplasmic and nuclear extracts were isolated as previously described (36). After stimulation, cells were washed with 1x PBS, and immediately scraped from the plate and centrifuged at 300 g for 5 min at 4°C. Pellets were resuspended in cytoplasmic isolation buffer [10 mM HEPES, 60 mM KCl, 1 mM EDTA, 0.075% (v/v) NP40, at pH 7.6, 1 mM DTT and 1% (v/v) protease/phosphates inhibitor] on ice, incubated for 10 min, and then centrifuged for 5 min at maximum speed (~14,000 g). The supernatant was transferred to a new pre-chilled tube containing 20% glycerol (cytoplasmic extract). The pellet was resuspended by vortex in nuclear isolation buffer [20 mM Tris Cl, 420 mM NaCl, 1.5 mM MgCl_2_, 0.2 mM EDTA, at pH 8.0, 25% (v/v) glycerol, and 1% (v/v) protease/phosphates inhibitor] and incubated on ice for 1 h with agitation every 15 min. Samples were then centrifuged at high speed for 15 min, and the supernatants were collected into a new pre-chilled tubes (nuclear extract). Both cytoplasmic and nuclear extracts were stored in −80°C till use. Complementary oligonucleotides for the STAT3 promoter probe containing ATF/CRE binding site was synthesized and biotinylated using biotin labeling Kit (Thermo Fisher #89818). EMSAs were performed using EMSA kit (Thermo Fisher #20148). In brief, STAT3 promoter probe was incubated with 10 μg of nuclear extracts from unstimulated or IL-22-stimulated CMT93 cells in the presence of poly deoxy-inosinic-deoxy-cytidylic acid and protein-DNA complexes were resolved by native polyacrylamide gel electrophoresis.

### Transmission electron microscopy (TEM)

Dissected and cleaned ileum tissues were washed with 1x HBSS, cut into small fragments (~0.2 cm), and fixed in 1x PBS with 4% PFA/2.5% Glutaraldehyde. Tissue processing and image acquisition were performed by the TEM Core facility at Institute of Cellular and Organism Biology, Academia Sinica, Taiwan.

### Statistical analysis

Statistical analysis was performed most of the time using Multiple T-test when comparing two populations (wild-type & knockout) for only one variable (body weight, colon length, survival, etc.). Two-way analysis of variance (ANOVA), comparing the mean differences between and within populations (wild-type & knockout) that have been split into two independent variables (un-stimulated/stimulated), was used for experiments of multiple statistical comparisons. Survival curve was calculated using the Kaplan–Meier method and statistical significance was calculated using Log rank (Mantel-Cox) test. GraphPad Prism 6 software was used to run the analysis. All the data are presented as mean with standard deviation, and *p* ≤ 0.05 was considered significant. The level of P value is expressed in asterisk as follow: ^*^P ≤ 0.05, ^**^P < 0.005, ^***^P < 0.0005.

## Results

### ATF3 is essential for balanced homeostasis in the intestine

**Figure 1:**
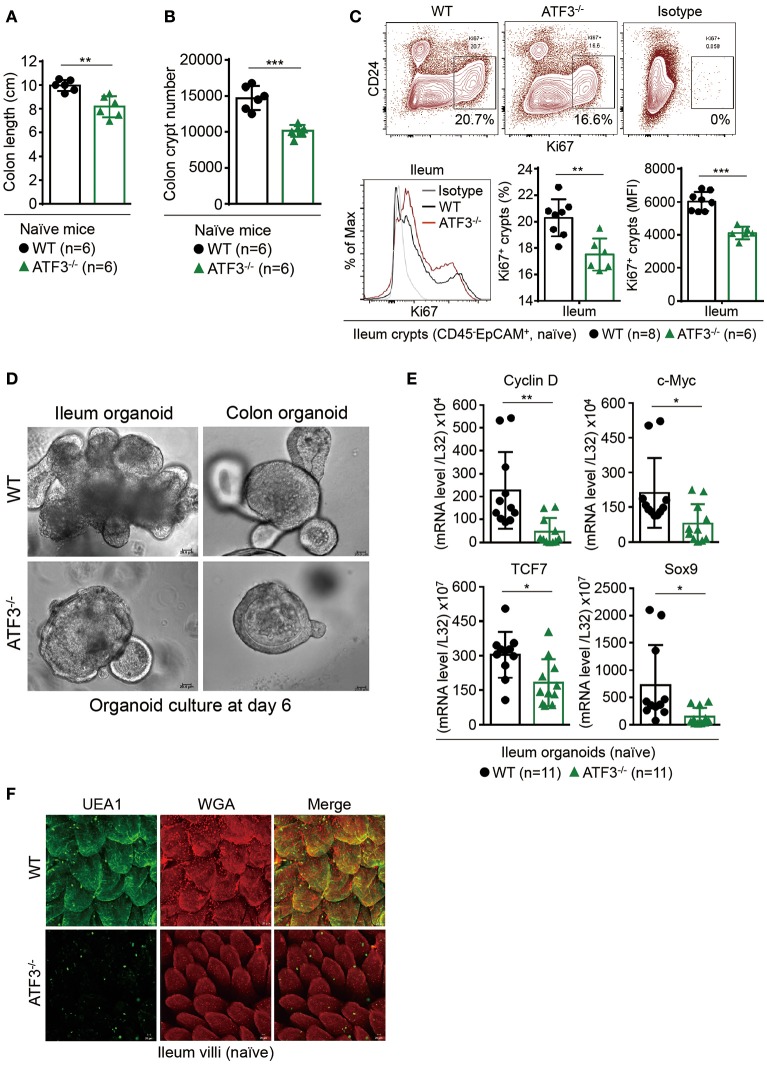
ATF3 maintains intestinal homeostasis. **(A)** Comparison of colon length between naïve mice as indicated. **(B)** Colon crypts from mice were isolated by shaking colon fragments in EDTA and counted under light microscopy. **(C)** Flow cytometry analysis of Ki67 and CD24 expression in ileum crypts, gated on the CD45^−^EpCAM^+^ populations, from the indicated naive mice. **(D)** Representative micrographs showing intestinal organoids derived from naïve mice. **(E)** Quantitative real-time PCR analysis of cell cycle genes in naïve ileum organoids at day 6 of culture (“n” indicates organoids derived from 4 mice each group). **(F)** Representative confocal images of whole mount tissues with co-immunofluorescence staining of UEA-1 and WGA in naïve ileum villi. Results were from at least two independent experiments and “n” refers to the number of mice unless indicated otherwise. All mice were at the age of 2~3 months old when analyzed. Statistical analysis was done using Multiple *T*-test on Prism software. ^*^P < 0.05, ^**^P < 0.005, ^***^P < 0.0005.

Given the fact that increased ATF3 level is associated with active IBD patients (21) and in order to better understand the function of ATF3 in the intestine, we first sought to determine the tissue distribution of ATF3 in more details. For this purpose, we prepared samples from the intestine of mice for mRNA analysis, including intestine fragments, villus samples by scraping off the surface of epithelial layer, and crypt samples by shaking fragments in the EDTA solution (Supplementary Figure 1A). We found ATF3 is more expressed in epithelial scrapes isolated from duodenum and jejunum, and is more abundant in ileum and colon crypts (Supplementary Figure 1B). Moreover, intestinal epithelial cells express higher mRNA levels of ATF3 than those tissues rich in immune cells including Peyer's Patches, mesenteric lymph nodes, inguinal lymph nodes, and spleen (Supplementary Figure 1B), suggesting that ATF3 has a more important regulatory role in the IECs. This finding prompted us to examine the characteristics of epithelial barrier integrity, determined macroscopically by the length of intestine, colon tissue histology, and the number of total crypts harvested from the intestine (30). Compared to wild-type littermates, we found that, at 3 months of age, naïve ATF3^−/−^ mice showed more shortened colon length, marked reduction in total crypt numbers (Figures 1A,B), slightly reduced body weight, less proliferative crypt cells, and increased cell infiltration in colon tissues (Supplementary Figures 1C,D), indicative of spontaneous inflammation in the colon. However, in ATF3^−/−^ mice, we did not observe any enlarged lymph nodes, splenomegaly or cellularity defects associated with other lymphoid organs at the steady state or during DSS colitis (data not shown).

Because most defects in ATF3^−/−^ mice were associated with epithelial barrier, we next examined the proliferation capacity of epithelial cells. In freshly isolated crypts, we found there is defective proliferation or regeneration of ileum or colon crypts by Ki67 staining (Figure 1C and Supplementary Figure 1E). To confirm this phenotypical abnormality is cell intrinsic to epithelial cells, we used primary organoid culture for further investigation. Intestinal organoid is an *ex vivo* primary epithelial culture fueled with epithelial stem cells and their differentiated daughter cells that can transform into a mini-intestine while maintaining all the physiological and pathological features of the tissue from which they are derived (37). Notably, ATF3^−/−^ organoids displayed slower growth rate, less budding (i.e., crypt formation), and less survival than wild-type organoids (Figure 1D). This growth impairment was also characterized by downregulation of the genes that control cellular proliferation and survival including Cyclin D, c-Myc, TCF7, and the stem cell marker Sox9 (Figure 1E). Together, we concluded ATF3 is essential for gut epithelial cell proliferation that directly controls crypt regeneration and barrier homeostasis.

Fucosylation is one of the most vital epithelial modifications involving adding oligosaccharides on glycoproteins or glycolipids. It plays a key role in regulating epithelial barrier, microbiota composition, and susceptibility to infection (38, 39). Epithelial fucosylation is catalyzed by fucosyltransferase enzymes, most notably the IBD risk gene fucosyltransferase 2 (*Fut2*) (5). We found that ileum fragments in naïve ATF3^−/−^ mice have decreased Fut2 mRNA expression compared to wild-type mice (Supplementary Figure 1F). It was shown fucosylated epithelial cells can be more specifically marked by whole mount tissue immunofluorescence co-staining of α(1,2)-fucose-recognizing lectin (ulex europaeus agglutinin-1 or UEA-1) and wheat germ agglutinin (WGA) (40). We therefore used UEA-1/WGA staining to further confirm FUT2 downregulation in ATF3-defiecitnt epithelial cells (Figure 1F). Since *Fut2* is one of IL-22-pSTAT3 regulated host defense genes (38), the lack of FUT2 expression and diminished UEA-1/WGA signals in ATF3^−/−^ IECs indicates that ATF3 is involved in epithelial IL-22-pSTAT3 fucosylation pathway which controls microbiota composition, host-commensal homeostasis and host defense against bacterial infection. Indeed, we found that ATF3^−/−^ mice were more susceptible to intestinal infection by Citrobacter rodentium (Supplementary Figure S2). Collectively, our results demonstrate that epithelial ATF3 is intrinsically essential for mucosal homeostasis in the intestine.

### Loss of ATF3 disrupts paneth cell homeostasis and stem cell regeneration

**Figure 2:**
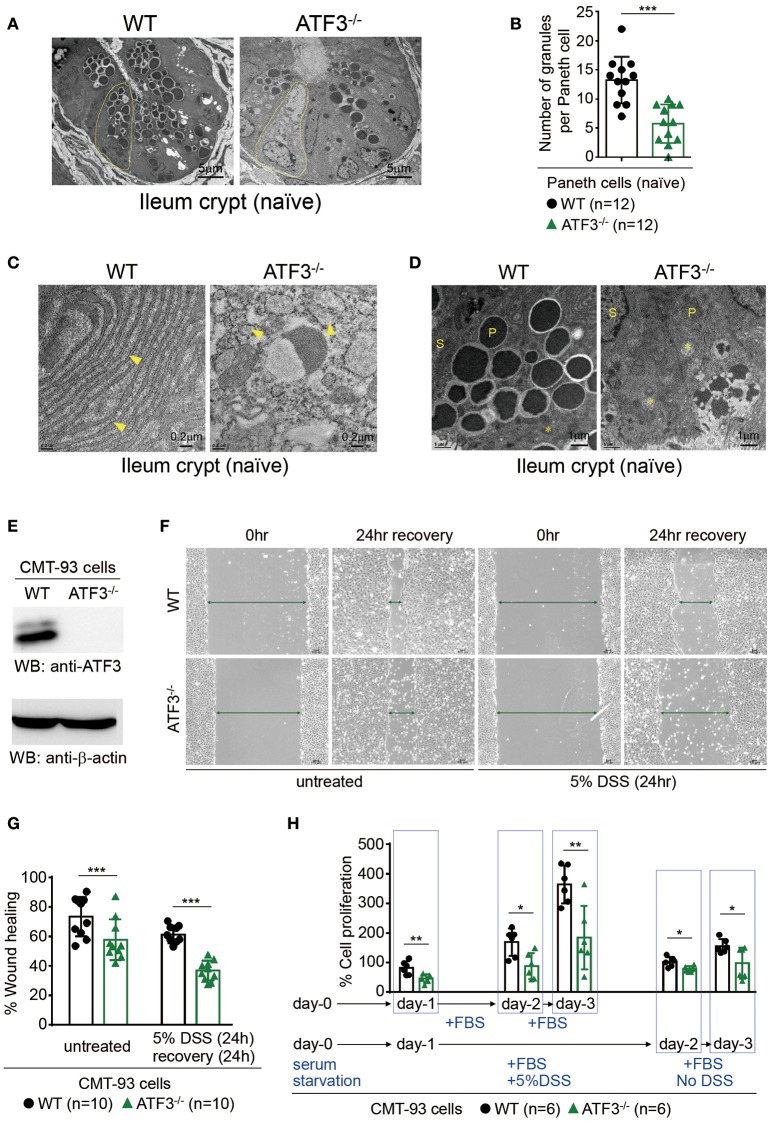
ATF3 regulates Paneth cell homeostasis and promotes wound healing and proliferation of epithelial cells. **(A–D)** Transmission electron microscopy images of naïve ileum crypts showing the structural appearance of Paneth cells. **(A)** Degeneration of Paneth cells (outlined in yellow) and loss of their apical granules in ATF3^−/−^ mice compared to wild-type mice. **(B)** Total number of granules in Paneth cells was counted manually in crypts (*n* = 12) imaged from 4 mice each group. **(C)** ER (indicated by arrowheads) fragmentation into cytoplasmic vesicles and (D) mitochondria (indicated by asterisks) swelling with damaged cristae in naïve ATF3^−/−^ mice were shown. P = Paneth cells, S = Stem cells. **(E–H)** Assays of wound healing and proliferation in CMT93 cells and “n” refers to number of CMT93 samples. **(E)** Western blot analysis of ATF3 ablation in CMT93 cells. **(F,G)** Wound healing assay of CMT93 cells with or without DSS treatment for injury. Wound was introduced by scratch and % of wound closure was normalized and calculated after 24 h of recovery. **(H)** Cell proliferation assay of CMT93 cells. Cells were serum starved, then proliferation was compared with or without injury introduced by DSS treatment as indicated. Total cell proliferation was expressed as percentage relative to the number of cells seeded. Results were representative of two independent experiments. Statistical analysis was done using Multiple *T*-test on Prism software. ^*^P < 0.05, ^**^P < 0.005, ^***^P < 0.0005.

Paneth cells, the maestros of epithelial anti-microbial immunity, are professional secretory cells with an apical clustering of secretory granules (41). Those granules within Paneth cells release key anti-microbial peptides (AMP) that help constitute a neat environment required for gut homeostasis (41). Because Paneth cells, together with stem cells, are located at the crypt base, the finding of compromised crypt homeostasis in ATF3^−/−^ mice prompted us to further examine whether ATF3 has a role in the development or immunity of Paneth cells. Transmission electron microscopy (TEM) of Paneth cells revealed a dramatic depletion, degeneration, and reduction in numbers of Paneth cells and their granules in ATF3^−/−^ mice (Figures 2A,B). Changes in inner cell structure and organization, such as fragmentation and disruption of the vacuolar system including endoplasmic reticulum (ER) and mitochondria, are hallmark morphological alterations occurring in apoptotic cells under TEM (42–44). Besides degeneration of Paneth secretory granules, fragmentation and abnormal distribution of ER, and swollen mitochondria with damaged cristae are typical morphological features of loss of cell homeostasis and cell death in Paneth/stem cell niche in ATF3^−/−^ mice (Figures 2C,D). Paneth cells cohabitate with stem cells at the crypt base forming a mutual niche of all the essential signals for stem cell maintenance (45). Therefore, any Paneth cell abnormality eventually leads to stem cell dysfunction, as evidenced by impaired cell survival, proliferation, and regeneration of ATF3^−/−^ IECs. This two-hit (Paneth/stem cells) abnormality phenomena due to ATF3 deficiency emphasizes the legitimacy of ATF3 to maintain the homeostasis and functionality of the crypt niche.

Next, we used a colon epithelial cell line, CMT93, to investigate whether ATF3 is involved in wound healing and epithelial regeneration in response to injury. Colitis-associated mucosal damage requires active and regulated processes of tissue healing and regeneration, which is facilitated by intestinal stem cells (ISCs). Accordingly, we performed wound healing assay in dextran sulfate sodium (DSS)-treated CMT93 cells, which mimics cell migration and tissue repair during regeneration *in vivo* after DSS-introduced epithelial damage. By monitoring wound closure after DSS exposure, we found that ATF3^−/−^ CMT93 cells reseal the open wound much slower compared to wild-type cells in both untreated and DSS-treated conditions (Figures 2E–G). Cell proliferation rate is a critical factor that contributes to barrier homeostasis. Notably, we found proliferation rate of ATF3^−/−^ CMT93 cells is significantly lower than wild-type cells in both untreated and DSS-treated conditions (Figure 2H), indicating that proliferative capability of ATF3^−/−^ epithelial cells was compromised, consistent to those results observed in organoid culture (Figures 1D,E). Collectively, we concluded ATF3 regulates barrier maintenance which involves epithelial cell regeneration and tissue repair.

### Epithelial ATF3 is protective during DSS-induced colitis

**Figure 3:**
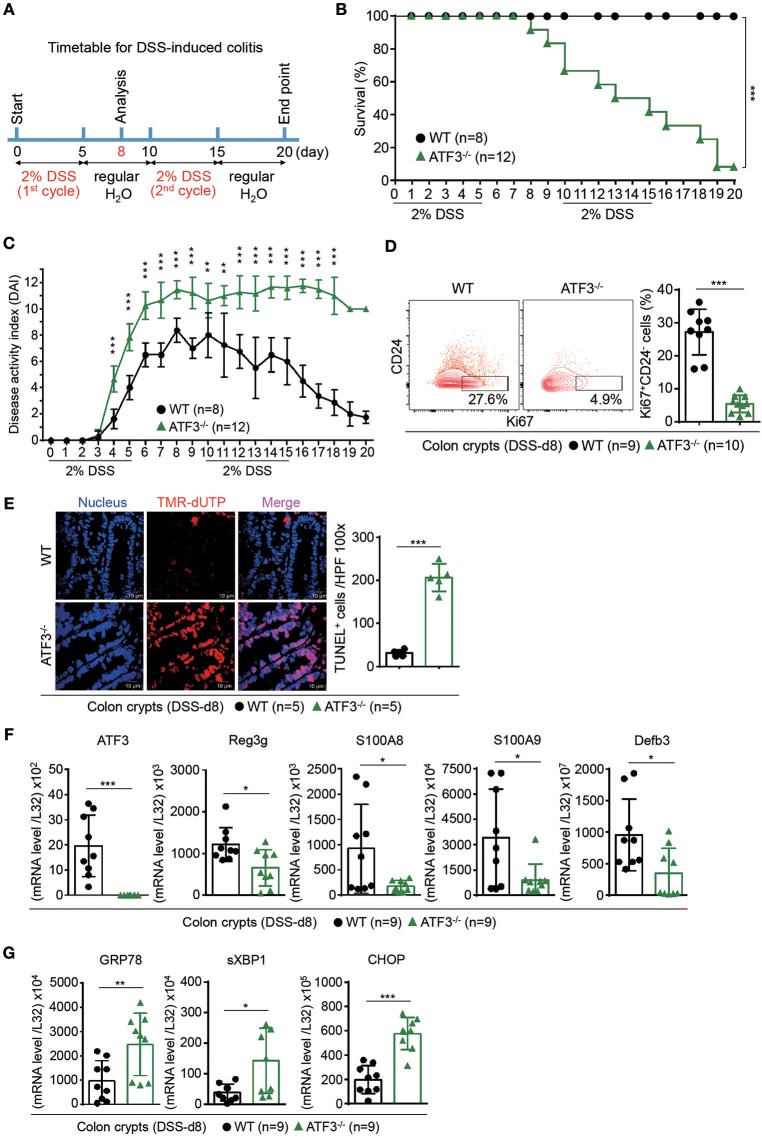
ATF3 protects mice from DSS-induced colitis. **(A)** Experimental protocol of DSS-induced colitis was shown. **(B)** Survival rate in mice after DSS treatment. **(C)** Disease activity index (DAI), a composite measurement of weight loss percentage, stool consistency, and blood in stools, was indicated in each group of mice during DSS colitis. **(D–G)** Analysis of colitis severity at day-8 post DSS treatment. **(D)** Flow cytometry of Ki67^+^ proliferating crypt cells in CD24^low/−^ cell population. **(E)** TUNEL assay showing apoptotic cells in colon tissues. Magenta positive apoptotic cells were quantified per 100x high-power field (HPF) from 10 different views of colon section from each mouse. **(F–G)** Quantitative real-time PCR analysis of crypt cells at day-8 post DSS. **(F)** Expression of ATF3 and anti-microbial peptide-related genes. **(G)** Expression of ER stress-related genes. Results were from two independent experiments. “n” refers to the number of mice analyzed. Survival curve was calculated using the Kaplan–Meier method and statistical significance was calculated using Log rank (Mantel-Cox) test. Statistical analysis was done using Multiple *T*-test on Prism software. ^*^P < 0.05, ^**^P < 0.005, ^***^P < 0.0005.

We next investigated whether disrupted Paneth/stem cell niche, due to loss of ATF3, will lead to clinical manifestation during intestinal stress such as inflammation. DSS-induced colitis was selected because it is well documented that DSS administration in mice causes epithelial barrier dysfunction which we believe ATF3 has a major regulatory role (46). Groups of wild-type and ATF3^−/−^ mice were given two cycles of 2% DSS in drinking water (Figure 3A), and monitored daily for disease activity index (DAI), by measuring body weight, stool consistency, and rectal bleeding as previously described (29). While naïve ATF3^−/−^ mice showed no signs of disease activity, notably, DSS-treated ATF3^−/−^ mice developed more severe colitis with lethal disease activity leading to reduced survival rate and much shortened colon length compared to wild-type mice (Figures 3B,C, and Supplementary Figures 3A,B). Since increased shortening of the colon during DSS colitis suggests impaired epithelial regeneration, we sacrificed mice for further analysis at day-8 after the first DSS cycle when DAI was highest in mice. Consistent to clinical manifestation, we found colon crypts are less proliferative by Ki67 staining in ATF3^−/−^ mice, which likely leads to decreased crypt numbers and increased colon tissue pathology (Figure 3D, and Supplementary Figure 3C–E). This indicates that wild-type mice can subdue DSS-induced inflammation by enhancing barrier repair through active epithelial cell proliferation, while ATF3^−/−^ mice exhibit fatal damage of epithelial barrier in terms of loss of cellular proliferation accompanied by increased apoptosis, as evidenced by increased TUNEL^+^ cells (Figure 3E). Collectively, the observations that DSS treatment in ATF3^−/−^ mice introduced more mucosal epithelial damage, extensive inflammation, loss of epithelial architecture, loss of crypts, and enhanced immune cell infiltrates, provide evidence that ATF3 is a pillar base for a balanced intestinal stress response to maintain mucosal integrity during colitis. Impaired AMP production and elevated levels of endoplasmic reticulum (ER) stress are believed to be prominently involved in the pathogenesis of IBD (41, 47). Notably, core intestinal AMPs, such as Reg3, defensin-β3, and the calcium binding proteins S100A8/A9, were markedly reduced in ATF3^−/−^ mice (Figure 3F). Abnormally active ER machinery, as evidenced by increased mRNA levels of ER stress-related genes including GRP78, sXBP1 and CHOP, was also detected in the colons of ATF3^−/−^ mice (Figure 3G). Taken together, we concluded ATF3^−/−^ mice suffer lethal colitis due to impaired epithelial regeneration and immunity, as well as enhanced cellular stress.

**Figure 4:**
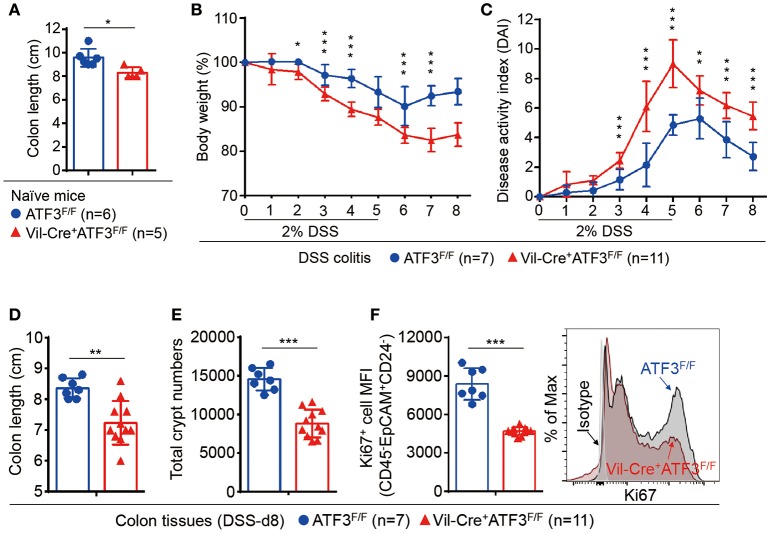
Epithelial ATF3 is required for protection against DSS colitis. **(A)** Comparison of colon length between 3-month-old naïve mice as indicated. **(B-F)** Analysis of colitis severity during DSS treatment. **(B)** Percentage of body weight loss during DSS colitis. **(C)** Disease activity index (weight loss percentage, stool consistency, and blood in stools) was indicated in each group of mice during DSS colitis. **(D)** Colon length, **(E)** total colon crypt numbers, and **(F)** Ki67^+^ proliferating crypt cells by flow cytometry analysis, were measured at day-8 post DSS treatment. Results were from two independent experiments. “n” refers to the number of mice analyzed. Statistical analysis was done using Multiple *T*-test on Prism software. ^*^P < 0.05, ^**^P < 0.005, ^***^P < 0.0005.

To further strengthen our conclusion that epithelial ATF3 mediates protection during DSS colitis, we obtained ATF3 flox mice and generated epithelium-specific ATF3 conditional (Vil-Cre^+^ATF3^F/F^) knockout mice (28, 48). Similar to global ATF3^−/−^ mice, at 3 months of age, naïve Vil-Cre^+^ATF3^F/F^ mice showed more shortened colon length and severe Paneth cell degeneration, compared to ATF3^F/F^ littermate mice (Figure 4A and data not shown). We found epithelium-specific ATF3 conditional knockout mice also recapitulate most phenotypes of DSS colitis observed in global ATF3^−/−^ mice, including increased disease activity index, more shortened colon length, reduced total crypt numbers and impaired epithelial regeneration (Figures 4B–F). Together, these results exclude the potential contribution of non-epithelial ATF3 from other cell types to colitis pathogenesis in our experimental model.

### Rectal organoid transfer INTO DSS-treated ATF3^−/−^ mice ameliorates colitis

**Figure 5:**
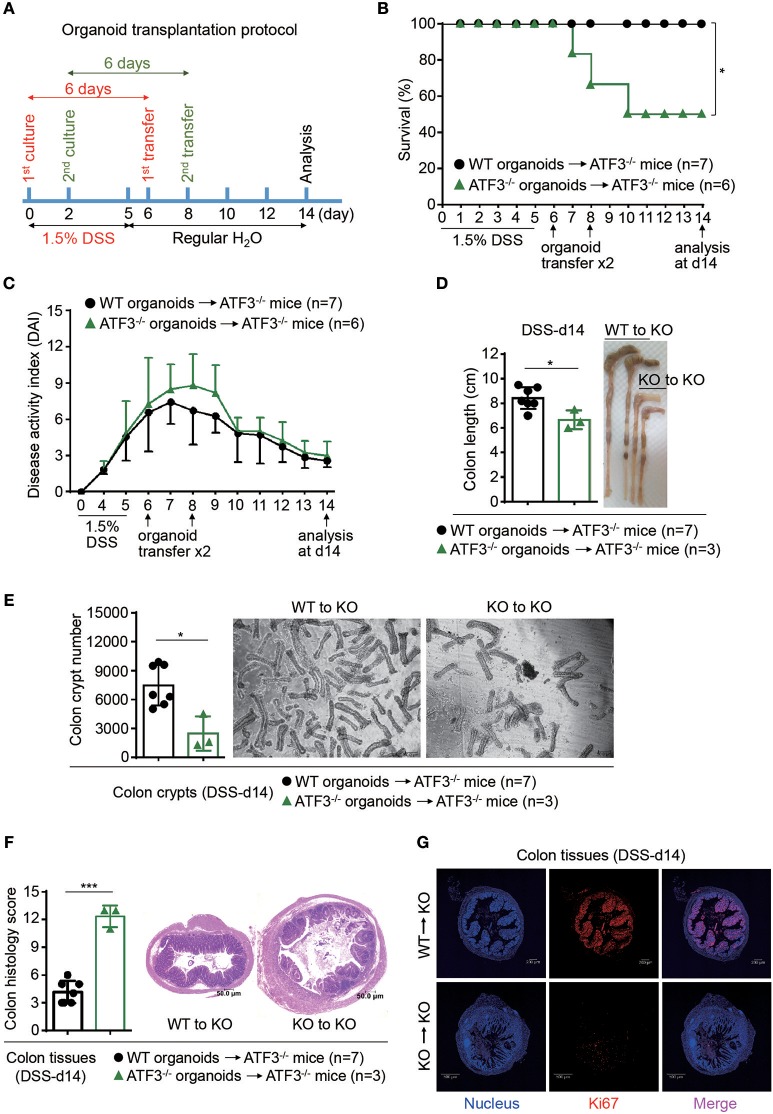
Rectal transplantation of wild-type organoids ameliorates colitis in ATF3-deficient mice. **(A)** Experimental protocol of organoid transfer post DSS colitis was shown. **(B–G)** Analysis of DSS colitis severity after organoid transfer. **(B)** Survival rate of mice after organoid transfer. **(C)** Disease activity index was indicated in each group of mice after organoid transfer. **(D)** Colon length, **(E)** total colon crypt numbers, **(F)** colon tissue histology scores (images were taken and scored at magnification of 10x) based on hematoxylin and eosin (H and E) staining, and **(G)** confocal images of colon tissues immunostained with the Ki67 proliferation marker at day-14 post DSS treatment were shown. Results were from two independent experiments. “n” refers to the number of mice analyzed. Survival curve was calculated using the Kaplan–Meier method and statistical significance was calculated using Log rank (Mantel-Cox) test. Statistical analysis was done using Multiple *T*-test on Prism software. ^*^P < 0.05, ^**^P < 0.005, ^***^P < 0.0005.

To determine whether ATF3-mediated protection during colitis is intrinsic or cell-autonomous to epithelial cells, rectal organoid transplantation after DSS induction of colitis was performed as previously described (33). Colon organoids, derived from wild-type or ATF3^−/−^ mice and cultured for 6 days, were used as donor cells (Figure 5A). Rectal transfer of donor organoids into ATF3^−/−^ recipient mice was performed at day-6 and day-8 post DSS treatment. Notably, ATF3^−/−^ recipient mice transplanted with wild-type, but not ATF3^−/−^ donor organoids, showed ameliorated inflammatory conditions, including increased survival and decreased disease activity (Figures 5B,C). At the day-14 after two organoid transfers, we found that ATF3^−/−^ mice receiving wild-type organoids recover from injury and improve colon integrity more rapidly, as evidenced by colon length and healthy appearance of both colon and cecum compared to those mice receiving ATF3^−/−^ organoids (Figure 5D). Correlated to this, total colon crypt numbers in mice receiving wild-type organoids was significantly higher than those of mice with ATF3^−/−^ organoid transfer (Figure 5E). Accordingly, colon pathology was decreased, likely due to improved tissue repair, in mice receiving wild-type organoids (Figures 5F,G). Together, these results indicate that restoring epithelial ATF3 signaling through wild-type organoid transplantation can effectively attenuate inflammatory conditions after DSS-induced colitis. These findings not only emphasize the protective role of intestinal epithelial barrier during colitis but also highlight the indispensable role for endogenous ATF3 in recovering and maintaining epithelial barrier functionality.

### ATF3 is a downstream target of IL-22 signaling and is required for IL-22-mediated AMP production

**Figure 6:**
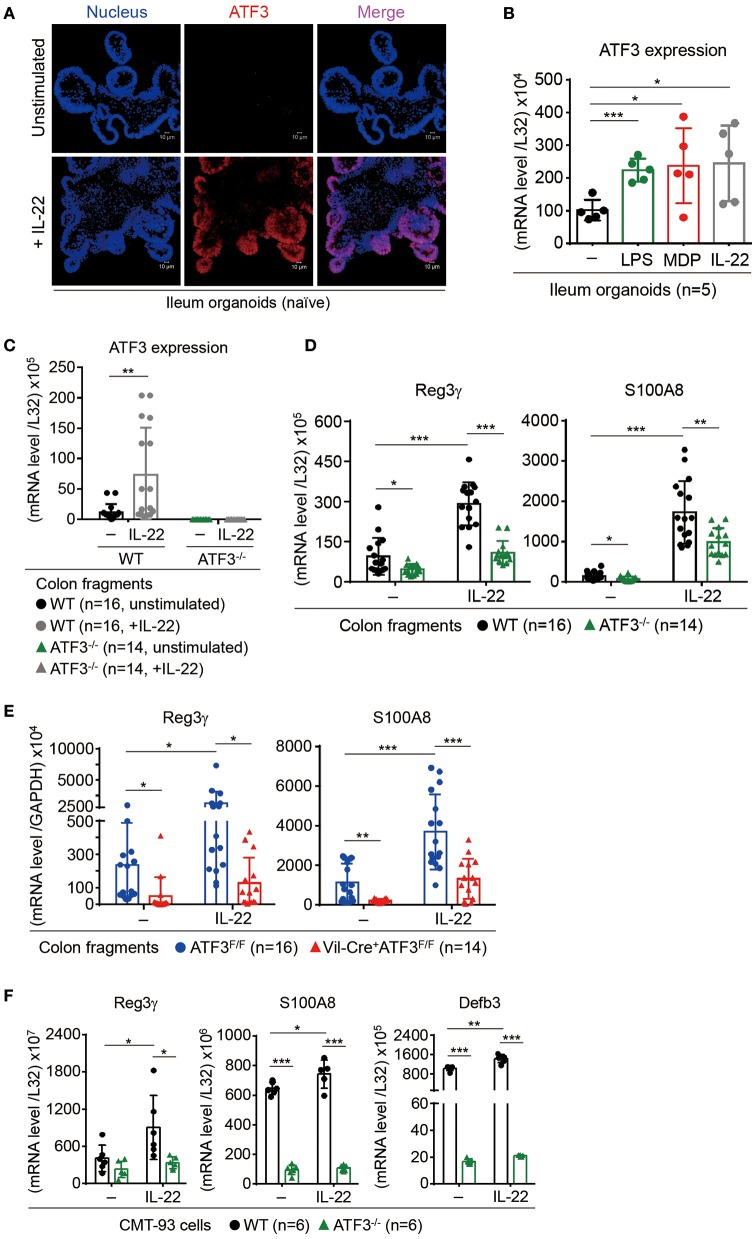
ATF3 mediates IL-22-induced production of anti-microbial peptides in epithelial cells. **(A,B)** Induction of ATF3 by IL-22 in organoids. **(A)** Representative three-dimensional confocal images of ATF3 expression in ileum organoids at day 6 of culture with or without IL-22 stimulation for overnight. **(B)** Quantitative real-time PCR analysis of ATF3 mRNA levels in ileum organoids at day 6 of culture, stimulated with lipopolysaccharide (LPS), muramyl dipeptide (MDP), or IL-22. “n” indicates organoids derived from 5 wild-type mice. **(C–E)** Quantitative real-time PCR analysis of genes in IL-22-stimulated colon fragments. Pieces of colon fragments (~0.5 cm) were cultured in complete DMEM with or without IL-22 for 5 h and mRNA levels of **(C)** ATF3, or **(D,E)** ant-microbial peptide Reg3γ and S100A8, were determined. “n” refers to the number of colon fragments obtained from 7 wild-type mice and 6 ATF3^−/−^ mice **(C,D)**, or 8 ATF3^F/F^ mice and 7 Vil-Cre^+^ATF3^F/F^ **(E)** mice. **(F)** Quantitative real-time PCR analysis of anti-microbial genes in IL-22-stimulated CMT93 cells. “n” refers to number of CMT93 samples analyzed. Results were from two independent experiments. Statistical analysis: Multiple *T*-test **B,C**, untreated control samples were used as the standard control for other stimulated samples), Two-way ANOVA test (**D–F**, for multiple comparison of samples). ^*^P < 0.05, ^**^P < 0.005, ^***^P < 0.0005.

In the intestine, IL-22 binds to IL-22 receptor (IL-22R) to transduce signaling exclusively in epithelial cells leading to STAT3 activation, epithelial proliferation and immunity (15). Direct stimulation of *ex vivo* cultured colon fragments with IL-22 induces AMP production that has been linked to host defense and protection of stem cell niche (11, 49). Nevertheless, the downstream IL-22-mediated genetic circuits are still largely unexplored. Although ATF3 is a stress-response molecule to extracellular stimuli such as DNA damage or Toll-like receptors (17), unexpectedly; we found ATF3 was upregulated in IL-22-stimulated ileum organoids or colon fragments (Figures 6A–C), indicating that ATF3 is a downstream target of IL-22 signaling. This finding also pinpoints the unique role of ATF3 in epithelial cells since IL-22R signaling is exclusive to epithelium in the gut. To further investigate whether ATF3 is functionally involved in IL-22 signaling, we analyzed the IL-22-induced AMP production in colon fragments, freshly isolated from wild-type or global ATF3^−/−^ mice (11). Notably, ATF3^−/−^ colon fragments, compared to wild-type colon fragments, showed reduced AMP production (Reg3γ and S100A8) after IL-22 stimulation (Figure 6D). To address concerns that other immune cells in ATF3^−/−^ colon fragments could also contribute to defective IL-22-induced AMP induction because of the use of global ATF3 knockout mice, we used colon fragments isolated from epithelium-specific ATF3 conditional knockout (Vil-Cre^+^ATF3^F/F^) mice or their littermates (ATF3^F/F^) for AMP production analysis and confirmed the results (Figure 6E). Similar observation was also obtained using CMT93 colon epithelial cells (Figure 6F). The defects of IL-22-induced AMP production in Vil-Cre^+^ATF3^F/F^ colon fragments further support why Vil-Cre^+^ATF3^F/F^ mice were also more susceptible to DSS-induced colitis compared to ATF3^F/F^ littermates (Figure 4). Taken together, we concluded that ATF3 is required to relay IL-22 signaling in epithelial cells for the induction of immunity and for epithelial regeneration as well, and that loss of ATF3 leads to compromised intestinal homeostasis and impaired recovery from mucosal damage due to lack of tissue-protective IL-22 signaling.

### ATF3 regulates IL-22-induced STAT3 phosphorylation via targeting phosphatases

**Figure 7:**
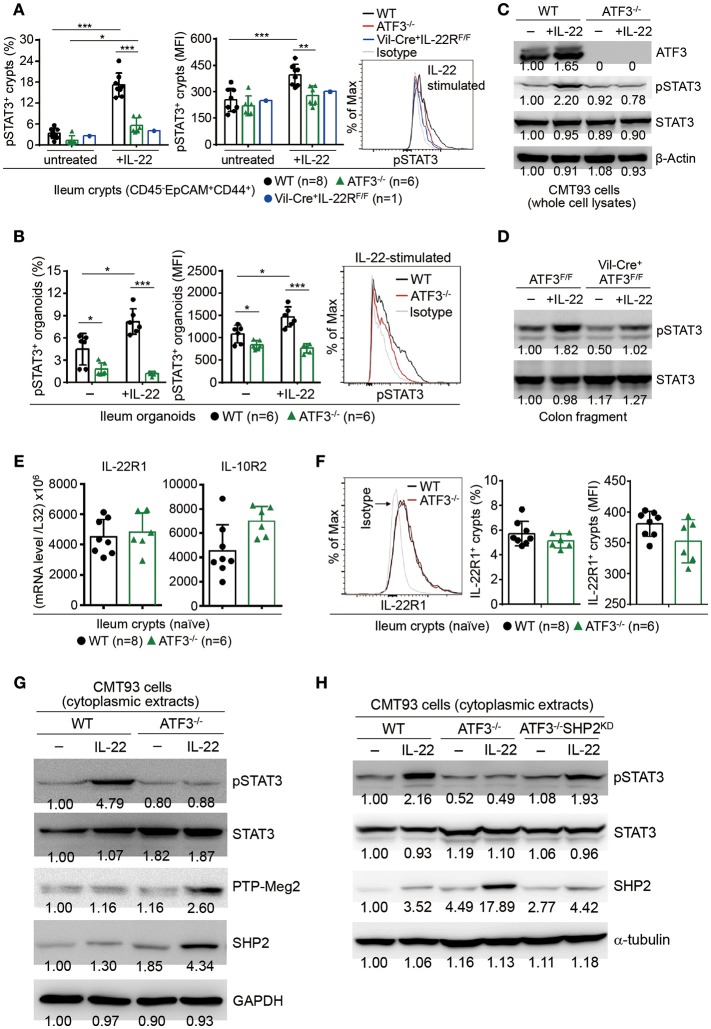
ATF3 promotes IL-22-induced STAT3 phosphorylation by suppressing phosphatases. **(A)** Freshly isolated ileum crypts, or **(B)** ileum organoids at day 6 of culture, were stimulated with IL-22, followed by fixation and intracellular staining of phospho-STAT3, and analyzed by flow cytometry. Western blot analysis of **(C)** IL-22-stimulated CMT93 cells, or **(D)** IL-22-stimuated colon fragments isolated from the indicated mice, for the expression of the indicated proteins. **(E)** Quantitative real-time PCR analysis of IL-22R1 and IL-10R2 mRNA levels in freshly isolated ileum crypts from mice. **(F)** Flow cytometry analysis of IL-22R1 in freshly isolated ileum crypt cells gated on the CD45^−^EpCAM^+^ population. **(G,H)** Western blot analysis of unstimulated or IL-22-stimulated CMT93 cells for the indicated proteins. ATF3^−/−^ CMT93 cells with SHP2 knockdown (ATF3^−/−^SHP2^KD^) were indicated. Images were representative of four independent experiments **(G–H)**. Results were from two independent experiments **(A–F)**. “n” refers to the number of mice analyzed **(A,B,E,F)**. Statistical analysis was done by multiple comparison in Two-way ANOVA test using Prism software. ^*^P < 0.05, ^**^P < 0.005, ^***^P < 0.0005.

In epithelial cells, STAT3 is the prime downstream target of IL-22 signaling and has been shown to be essential for intestinal organoid growth (7). IL-22 induces STAT3 phosphorylation on tyrosine-705, then subsequent dimerization and nuclear translocation of phosphorylated STAT3 initiate its transcriptional activity for programming sets of genes associated with AMP production, cell proliferation, tissue repair, and survival (8, 15). Mutations in STAT3 have been identified as susceptibility factors for IBD and loss of epithelial STAT3 rendered mice more susceptible to DSS colitis (9, 50, 51). Given that the expression of AMP such as Reg3β/γ is dependent on STAT3 signaling (9), we reasoned that ATF3-mediated AMP induction during IL-22 signaling might be through an action on STAT3 activation. Indeed, we found IL-22-induced STAT3 phosphorylation is dramatically abolished in the absence of ATF3, in freshly isolated ileum crypts, cultured ileum organoids, or CMT93 cells (Figures 7A–C). We further confirmed that IL-22-induced, ATF3-mediated STAT3 activation is epithelium-specific (Figure 7D). In addition, decreased IL-22 signaling was not due to downregulation of the IL-22 receptor complex, composed of IL-22R1 and IL-10R2 subunits (Figures 7E,F), in ATF3^−/−^ epithelial cells, indicative of an intrinsic cellular defect.

We next sought to determine whether ATF3, as a transcription factor, can directly bind to the STAT3 promoter to regulate its expression or activation during IL-22 signaling. Biotinylated DNA probe containing ATF/CRE binding site within the STAT3 promoter was used for the electrophoretic mobility shift assay (EMSA) (Supplementary Figure 4A). Notably, we were not able to detect a shift of the DNA-protein complex consisting of nuclear ATF3 and the STAT3 promoter probe (Supplementary Figure 4B) (52), indicating that ATF3 does not target STAT3 directly during IL-22 signaling in epithelial cells. We then explored the possibility whether ATF3 targets negative regulators of STAT3, such as protein tyrosine phosphatases (PTPs), to promote STAT3 activation (53). We tested several PTPs and identified two PTPs, SHP2 and PTP-Meg2, both known to dephosphorylate STAT3 (54, 55). Notably, loss of ATF3 in CMT93 cells led to increased levels of SHP2 and PTP-Meg2 at the steady state and much higher levels after IL-22 stimulation (Figure 7G), suggesting ATF3 negatively regulates these PTPs. To provide evidence that PTPs are upstream regulators of STAT3 phosphorylation during IL-22-ATF3 signaling, SHP2 was knockdown (SHP2^KD^) by shRNA in ATF3^−/−^ cells (namely ATF3^−/−^SHP2^KD^). Notably, we found that impaired STAT3 phosphorylation in ATF3^−/−^ cells was comparably restored in ATF3^−/−^SHP2^KD^ cells (Figure 7H), indicating that SHP2 is acting upstream of STAT3 as an inhibitor via de-phosphorylation in gut epithelial cells (53). Taken together, we concluded that ATF3 functions as a repressor to inhibit PTPs such that those PTPs are not functionally targeting STAT3 for suppression.

### ATF3 targets intestinal Th17 cell functionality via IL-6-PSTAT3 signaling

**Figure 8:**
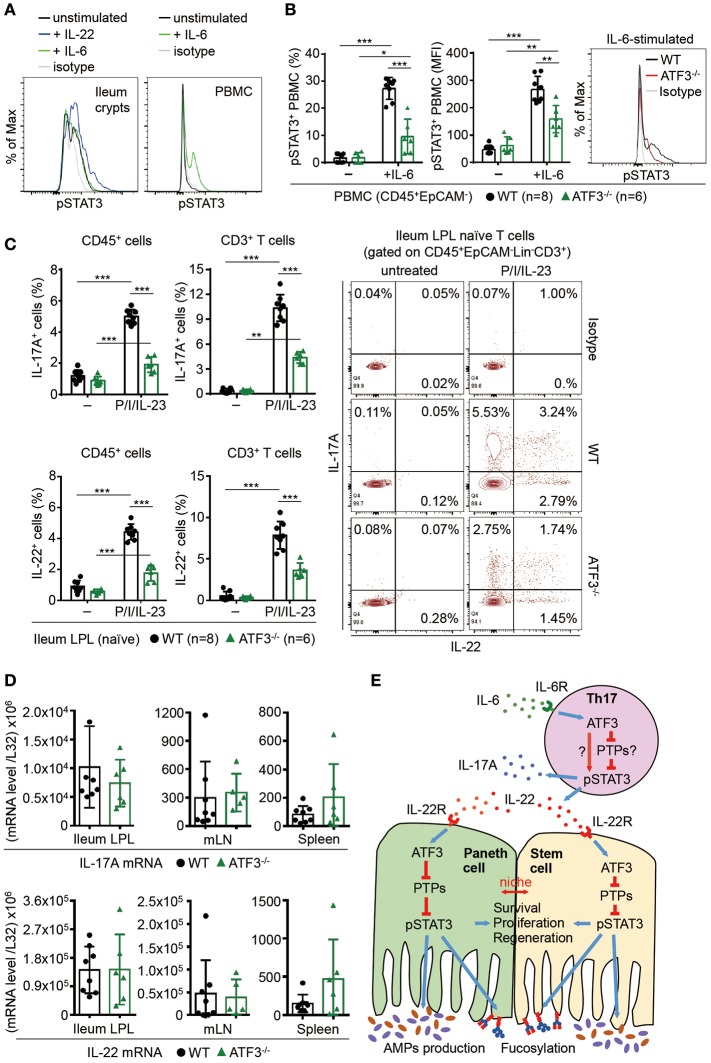
ATF3 regulates IL-6-pSTAT3 signaling in intestinal Th17 cells. Flow cytometry analysis of phospho-STAT3 in **(A)** IL-6 or IL-22 stimulated freshly isolated ileum crypts or IL-6-stimulated peripheral blood mononuclear cell (PBMC) from wild-type mice, or in **(B)** IL-6-stimulated PBMC from wild-type and ATF3-deficient mice. **(C)** Flow cytometry analysis of intracellular IL-17A and IL-22 expression in naïve lamina propria T cells from the indicated mice. Cells were treated with PMA, ionomycin and IL-23 in the presence of BFA for 4 h before analysis and gated on live CD45^+^EpCAM^−^Lin^−^CD3^+^ population as shown. **(D)** Quantitative real-time PCR analysis of IL-17A and IL-22 mRNA levels in freshly isolated lamina propria (LPL) cells, mesenteric lymph nodes (mLN), or splenocytes. **(E)** Model of ATF3-mediated mucosal immunity via cross-regulation between IL-22-pSTAT3 signaling in epithelium (associated with AMP production and epithelial fucosylation) and IL-6-pSTAT3 signaling in Th17 cells (associated with signature IL-17A and IL-22 production). “n” refers to the number of mice analyzed. Statistical analysis was done by multiple comparison in Two-way ANOVA test using Prism software. ^*^P < 0.05, ^**^P < 0.005, ^***^P < 0.0005.

Because IL-6 could be produced by epithelial cells and it is also a strong STAT3 inducer, particularly in the context of inflammation (56, 57), we first examined whether IL-6 activates STAT3 in freshly isolated epithelial cells. Consistent to a study showing that loss of IL-6 did not affect epithelial STAT3 phosphorylation (9), we found IL-6 does not activate STAT3 in gut epithelial cells (Figure 8A). Intriguingly, IL-6 induces STAT3 activation in CD45^+^ mononuclear cells and we found ATF3 also positively regulates IL-6-induced STAT3 activation in CD45^+^ cells (Figures 8A,B). A recent study reported that in the gut, the dual-specificity phosphatase 2 (DUSP2) targets and catalyzes STAT3 de-phosphorylation leading to impaired Th17 cell development (58). Therefore, given that IL-6 drives Th17 cell differentiation whose regulation is tightly linked to IBD pathogenesis (1, 59), we next investigated whether loss of ATF3 in mice affects intestinal Th17 cell development. Notably, while mRNA and protein levels of both IL-22 and IL-17A were not much changed at the steady state, the capability of lamina propria Th17 cells to produce IL-22 or IL-17A, after ionomycin and PMA stimulation, was significantly compromised in ATF3^−/−^ cells (Figures 8C,D). In addition, we found in ATF3^−/−^ mice that mRNA levels of IL-6, IL-6R1, and gp130 (the signaling subunit of the IL-6 receptor complex) were not altered in various intestinal tissues (Supplementary Figure 5), excluding the possibility that compromised Th17 accumulation in the gut was due to decreased IL-6 or IL-6 receptor complex. Because increased expression of IL-6 and IL-22 are associated with IBD in patients (60, 61), our findings here pinpoint a cross-regulation of ATF3 for intestinal immunity maintenance, between IL-22-induced STAT3 activation in gut epithelial cells and IL-6-induced STAT3 activation in CD45^+^ cells especially Th17 cells (summarized in Figure 8E).

## Discussion

In light of a recent microarray analysis showing up-regulation of ATF3 in patients with active IBD (21), while lacking evidence supporting a role for ATF3 in intestinal homeostasis and IBD pathogenesis, we have performed in-depth analyses here, using primary intestinal organoids and animal models, to reveal how ATF3 is acting as a critical downstream regulator of the IL-22 signaling cascade in intestinal epithelial cells. We further identified a novel IL-22-mediated circuit in epithelial cells where ATF3 relays IL-22 signaling to inhibit PTPs to prevent them from inactivating STAT3 phosphorylation. Given that IL-22 itself could also induce ATF3, our results therefore illustrate a delicate mechanism where ATF3 facilitates functionality and amplification of IL-22 signaling. In addition, we provide evidence that ATF3 influences IL-6-mediated STAT3 activation which might be involved in intestinal Th17 accumulation, survival or development. As both IL-6 and IL-22 controls STAT3 activation which is associated with cell homeostasis, host defense, inflammation and tumorigenesis (56), our findings of ATF3-mediated cross-regulation between lymphoid cells and epithelial cells in the intestine support ATF3 as a novel and critical gatekeeper for intestinal immunity.

The intestinal barrier is composed of a versatile and dynamic layer of epithelial cells which is, to a great extent, maintained by a niche between intestinal stem cells and Paneth cells located at the crypt base (45, 62). Intriguingly, loss of ATF3 disrupts this niche leading to decreased Ki67^+^ proliferating transit-amplifying (TA) cells and total crypt numbers (i.e., regeneration capability) at the steady state or during colitis. The disrupted Paneth/stem cell homeostasis is likely due to compromised IL-22-induced STAT3 activation in ATF3^−/−^ stem cells, as is also supported by a recent study showing IL-22 activates STAT3 signaling in intestinal organoids and promotes stem cell regeneration (7). Notably, while IL-22 does not enhance STAT3 phosphorylation in Paneth cells (7), we found that ATF3 deficiency *in vivo* leads to Paneth cell degeneration featured by loss of AMP-producing granules. Although genetic depletion of Paneth cells *in vivo* results in the concomitant loss of Lgr5^+^ stem cells (45), we reason that ATF3 is primarily targeting stem cell homeostasis and regeneration, but not Paneth cells in the niche, as organoid transplantation effectively ameliorates colitis and restores total crypt numbers in DSS-treated ATF3^−/−^ mice. Thus, at the cellular level, ATF3-regulated epithelial niche homeostasis (but not ATF3-regulated Th17 cell functionality, see below) primarily determines intestinal immunity and susceptibility to colitis, while at the molecular level, ATF3-regulated IL-22-STAT3 activation determines full functionality of intestinal stem cells.

Clinical relevance of IL-22 and IL-6 signaling to IBD pathogenesis has been established (1, 10, 26, 61). STAT3 activation could be induced by IL-6R, IL-23R, or IL-22R signaling in Th17 cells (for IL-6R, IL-23R) and epithelial cells (for IL-22R) (1, 9, 56, 63). Components within these three signaling pathways are mostly overlapping and associated with IBD signaling modules by genome-wide association studies (1, 51). While we revealed a role for ATF3 in the IBD gene network of IL-22-STAT3, a function for ATF3 in IL-6-STAT3 or even IL-23-STAT3 activation in Th17 cell network has also been observed in our study. As an upstream regulator, ATF3 could suppress IL-6 transcription or even IL-22 transcription via NF-kB (18, 64). However, as a downstream regulator, we showed ATF3 is required to relay IL-6 and IL-22 signaling for the induction of STAT3 phosphorylation. Intriguingly, loss of ATF3 *in vivo* appears to compromise epithelial function via IL-22-ATF3-STAT3 and impair Th17 function via IL-6-ATF3-STAT3, without affecting overall levels of IL-6 and IL-22 in the gut. Thus, ATF3 seems to play a more dominant role in the downstream of IL-22 signaling circuit *in vivo*, compared to a role in IL-6 signaling, as global ATF3^−/−^ mice were more susceptible to DSS colitis and Citrobacter infection. In light of this cross-regulation by ATF3 in different cell types, more dedicated genetic studies remain needed to untangle complicated ATF3-mediated function in intestinal cell activation and disease pathogenesis.

Toll-like receptor 4 (TLR4) signaling by LPS in macrophages induces ATF3-mediated IL-6 suppression via ATF3 binding to the ATF/CRE site of STAT3 promoter in the IL-6 gene (18). Binding of ATF3 to the STAT3 promoter for gene inactivation has also been shown in human hepatocellular carcinoma (25). However, we found ATF3 itself does not bind to the STAT3 promoter directly, at least in CMT93 epithelial cells, excluding the possibility of direct suppression of STAT3 by ATF3. Protein tyrosine phosphatases (PTPs) are known negative regulators of STAT3 signaling (53). A recent study showed phosphatase DUSP2 (PAC1) interacts with STAT3 and catalyzes de-phosphorylation of STAT3 (58). During our screening of PTPs for potential ATF3 targets, we found loss of ATF3 in CMT93 cells does not affect DUSP2 levels (data not shown), while levels of PTP-SHP2 and PTP-Meg2 are increased. Although restoration of STAT3 phosphorylation in ATF3^−/−^SHP2^KD^ cells confirms IL-22-induced, ATF3-mediated SHP2 is acting upstream of STAT3 in epithelial cells, the same signaling cascade (i.e., IL-6-ATF3-PTP-STAT3) was not validated in IL-6-induced, ATF3-mediated STAT3 activation in T cells. Whether ATF3 targets STAT3 directly, or indirectly via PTPs in Th17 cells, needs further investigation, as requirement for transcriptional regulation by ATF3 is cell type-dependent. Collectively, our study here revealed unique and protective roles of ATF3 in intestinal immunity, where ATF3 links IL-22 signaling to STAT3 activation via targeting PTPs for epithelial immunity, while ATF3 links IL-6 signaling to STAT3 activation for Th17-mediated immunity.

## Ethics statement

John T. Kung, Ph.D., IACUC Chair, The Institutional Animal Care and Use Committee of Academia Sinica All animal studies described in the manuscript were approved and adhered to the guidelines and policies of the Institutional Animal Care and Use Committee of Academia Sinica (AS IACUC). *In vivo* animal studies were designed and performed based on the 3R (to replace, to reduce, to refine) principles and animal rights and welfare defined by the IACUC of Academia Sinica.

For experimental ethics, the authors claimed all data reported in the manuscript has no research misconduct, including fabricating or falsifying the results, plagiarism, or data duplication. We agree all original results described in the manuscript, once published, would be open for data sharing to the public. Conduct of research is in full compliance to the guidelines and policies of the Institution of Biomedical Sciences, Academia Sinica in Taiwan.

## Author contributions

DG designed, performed the experiments, analyzed the data, and wrote the manuscript. JS, M-CL, C-FC, and J-WS helped in generating ideas, designing the experiments, and troubleshooting for this study. H-HL and H-YC helped in designing and performing critical experiments for revised manuscript. Y-CL helped mouse husbandry and genotyping. J-WS wrote the manuscript and did the critical revision of the manuscript.

### Conflict of interest statement

The authors declare that the research was conducted in the absence of any commercial or financial relationships that could be construed as a potential conflict of interest.

## Supplementary material

Supplementary Figure 1 ATF3 regulates intestinal homeostasis. **(A)** Isolation of ileum villi by scraping and crypts by fragment shaking in 30 mM EDTA for 30 min. **(B)** Quantitative real-time PCR analysis of ATF3 mRNA levels in freshly isolated tissues from different intestinal compartments and abdominal organs. **(C)** Comparison of body weight of the indicated mice at the age of 2~3 months old. **(D)** Flow cytometry analysis of Ki67 and CD24 expression in colon crypts, gated on the CD45^−^EpCAM^+^ populations, from the indicated naive mice. **(E)** H&E sections of naïve colon tissues showing healthy colon structure in WT mice while increased cell infiltration in ATF3-deficient colon. **(F)** Quantitative real-time PCR analysis of Fut2 mRNA levels in naïve ileum fragments (“n” refers to the number of fragments obtained from 7 wild-type mice and 7 ATF3^−/−^ mice). Results were from two independent experiments and “n” refers to the number of mice **(B–D)**, unless indicated otherwise. Statistical analysis was done using Multiple *T*-test on Prism software. ^*^*P* < 0.05, ^**^*P* < 0.005, ^***^*P* < 0.0005.

Supplementary Figure 2 ATF3^−/−^ mice were more susceptible to Citrobacter infection. Groups of mice were infected with a single dose (8 × 10^8^ CFU) of Citrobacter rodentium by oral gavage. **(A)** Fecal colony-forming unit (CFU) was measured and compared at the indicated days post Citrobacter infection. **(B)** Colonoscopy view showing ulceration/bleeding in the colon of ATF3^−/−^ mice at day 7 (Citro-d7) post infection. **(C)** Colon CFU and **(D)** colon length at day 12 post infection were measured and compared. Results were representative of two independent experiments. “n” refers to the number of mice used for analysis. Statistical analysis was done using Multiple *T*-test on Prism software. ^*^*P* < 0.05, ^**^*P* < 0.005.

Supplementary Figure 3 ATF3^−/−^ mice were more susceptible to DSS colitis. Analysis of colitis severity during DSS treatment. **(A)** Percentage of body weight loss during DSS colitis. **(B)** Colon length, **(C)** total colon crypt numbers, **(D)** colon tissue histology scores based on hematoxylin and eosin (H and E) staining, and **(E)** colonoscopic appearance were analyzed at the indicated day post DSS treatment. Results shown were from two independent experiments and “n” refers to the number of mice used for analysis. Statistical analysis was done using Multiple *T*-test on Prism software. ^*^*P* < 0.05, ^**^*P* < 0.005, ^***^*P* < 0.0005.

Supplementary Figure 4 ATF3 does not target the STAT3 promoter during IL-22 signaling in CMT93 epithelial cells. **(A)** Sequence of the mouse STAT3 promoter. Oligonucleotide probe (underlined), containing ATF/CRE binding site (shown in red) and STAT-binding element (SBE, shown in green) in the STAT3 promoter, was used for EMSA experiment. CTG (indicated in purple) is the transcriptional initiation site. GC box (shown in blue) is indicated. **(B)** EMSA assay, control system: Lane #1, only biotin-labeled 60 bp duplex bearing the EBNA-1 binding sequence showing only free DNA. Lane #2, biotin-labeled 60 bp duplex bearing the EBNA-1 binding sequence and EBNA extract showing DNA-protein complex shift. In assay with CMT93 cells, EMSA was performed with biotinylated STAT3 promoter probe and nuclear extracts prepared from WT or ATF3^−/−^ CMT93 cells with or without IL-22 stimulation (50 ng/ml, 10 min after 5 h of serum starvation). EBNA: Epstein-Barr Nuclear Antigen. Results shown were representative of two independent experiments.

Supplementary Figure 5 ATF3 deficiency in mice does not affect mRNA levels of IL-6, IL-6R1 and gp130 in intestinal compartments. Quantitative real-time PCR analysis of **(A)** IL-6, **(B)** IL-6R1, and **(C)** gp130 mRNA levels in freshly isolated tissues from different intestinal compartments and abdominal organs. Samples of mesenteric lymph nodes (mLN) and spleen were used for comparison. Results shown were combined from two independent experiments and “n” refers to the number of mice used for analysis. No statistical difference between wild-type and ATF3^−/−^ mice was detected.
