# Supplementary figures and images for: ATF3 Sustains IL-22-Induced STAT3 Phosphorylation to Maintain Mucosal Immunity Through Inhibiting Phosphatases
Source: Front Immunol. 2018 Nov 5;9:2522. doi: 10.3389/fimmu.2018.02522 (PMC6230592; doi:10.3389/fimmu.2018.02522)

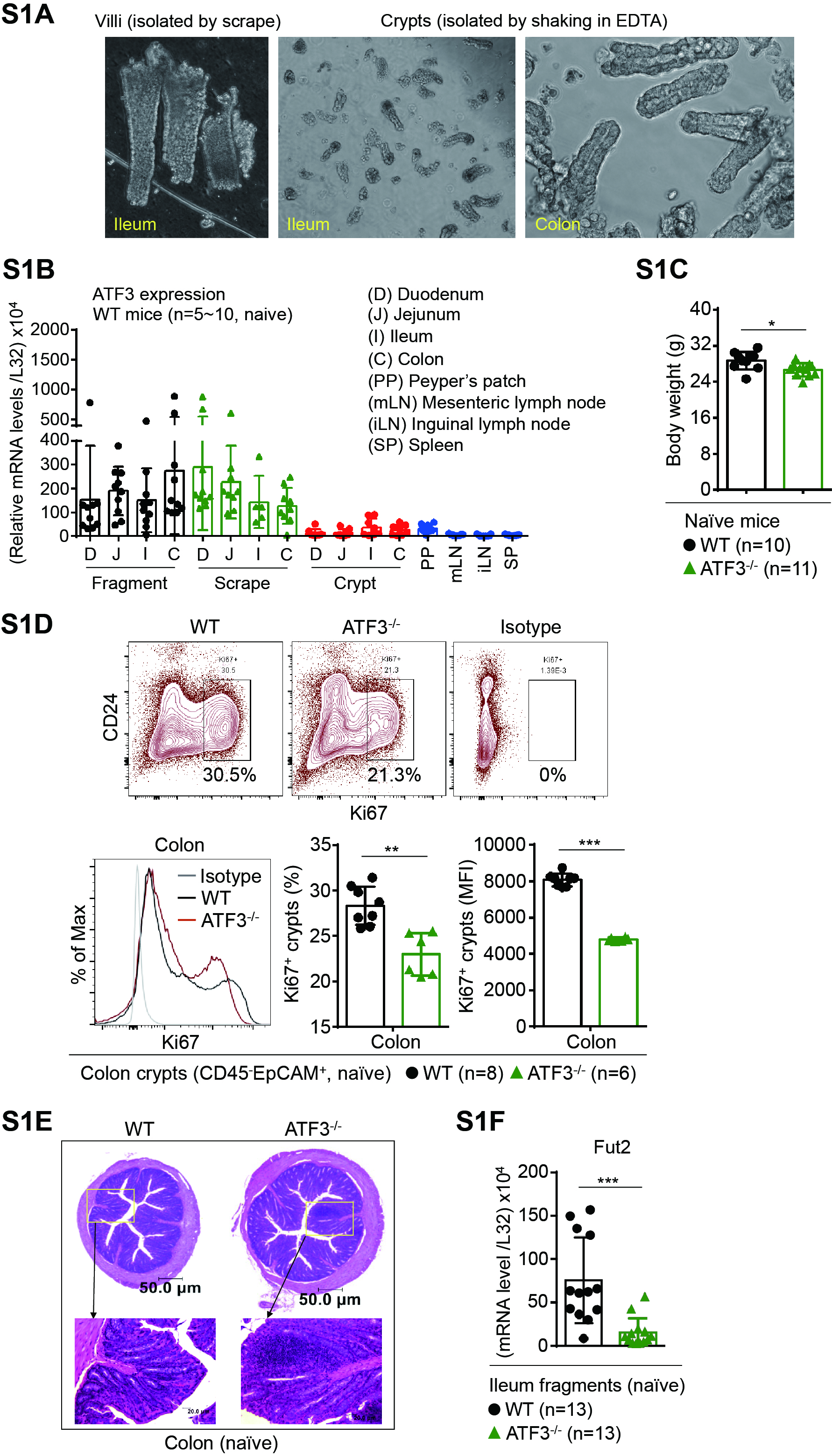

Supplement: Supplementary Figure 1 — ATF3 regulates intestinal homeostasis. (A) Isolation of ileum villi by scraping and crypts by fragment shaking in 30 mM EDTA for 30 min. (B) Quantitative real-time PCR analysis of ATF3 mRNA levels in freshly isolated tissues from different intestinal compartments and abdominal organs. (C) Comparison of body weight of the indicated mice at the age of 2~3 months old. (D) Flow cytometry analysis of Ki67 and CD24 expression in colon crypts, gated on the CD45−EpCAM+ populations, from the indicated naive mice. (E) H&E sections of naïve colon tissues showing healthy colon structure in WT mice while increased cell infiltration in ATF3-deficient colon. (F) Quantitative real-time PCR analysis of Fut2 mRNA levels in naïve ileum fragments (“n” refers to the number of fragments obtained from 7 wild-type mice and 7 ATF3−/− mice). Results were from two independent experiments and “n” refers to the number of mice (B–D), unless indicated otherwise. Statistical analysis was done using Multiple T-test on Prism software. *P < 0.05, **P < 0.005, ***P < 0.0005. [file Image_1.JPEG]

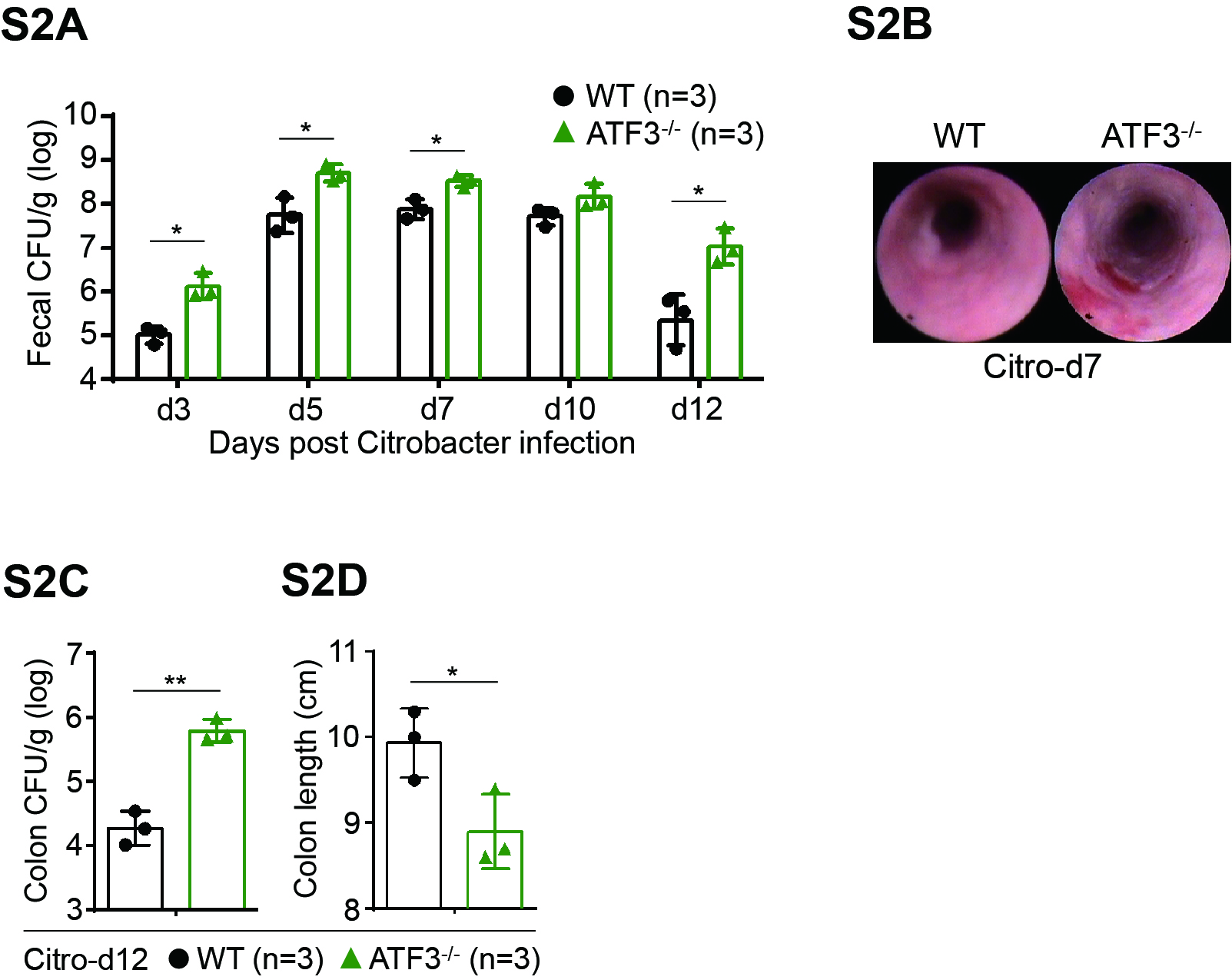

Supplement: Supplementary Figure 2 — ATF3−/− mice were more susceptible to Citrobacter infection. Groups of mice were infected with a single dose (8 × 10^8^ CFU) of Citrobacter rodentium by oral gavage. (A) Fecal colony-forming unit (CFU) was measured and compared at the indicated days post Citrobacter infection. (B) Colonoscopy view showing ulceration/bleeding in the colon of ATF3−/− mice at day 7 (Citro-d7) post infection. (C) Colon CFU and (D) colon length at day 12 post infection were measured and compared. Results were representative of two independent experiments. “n” refers to the number of mice used for analysis. Statistical analysis was done using Multiple T-test on Prism software. *P < 0.05, **P < 0.005. [file Image_2.JPEG]

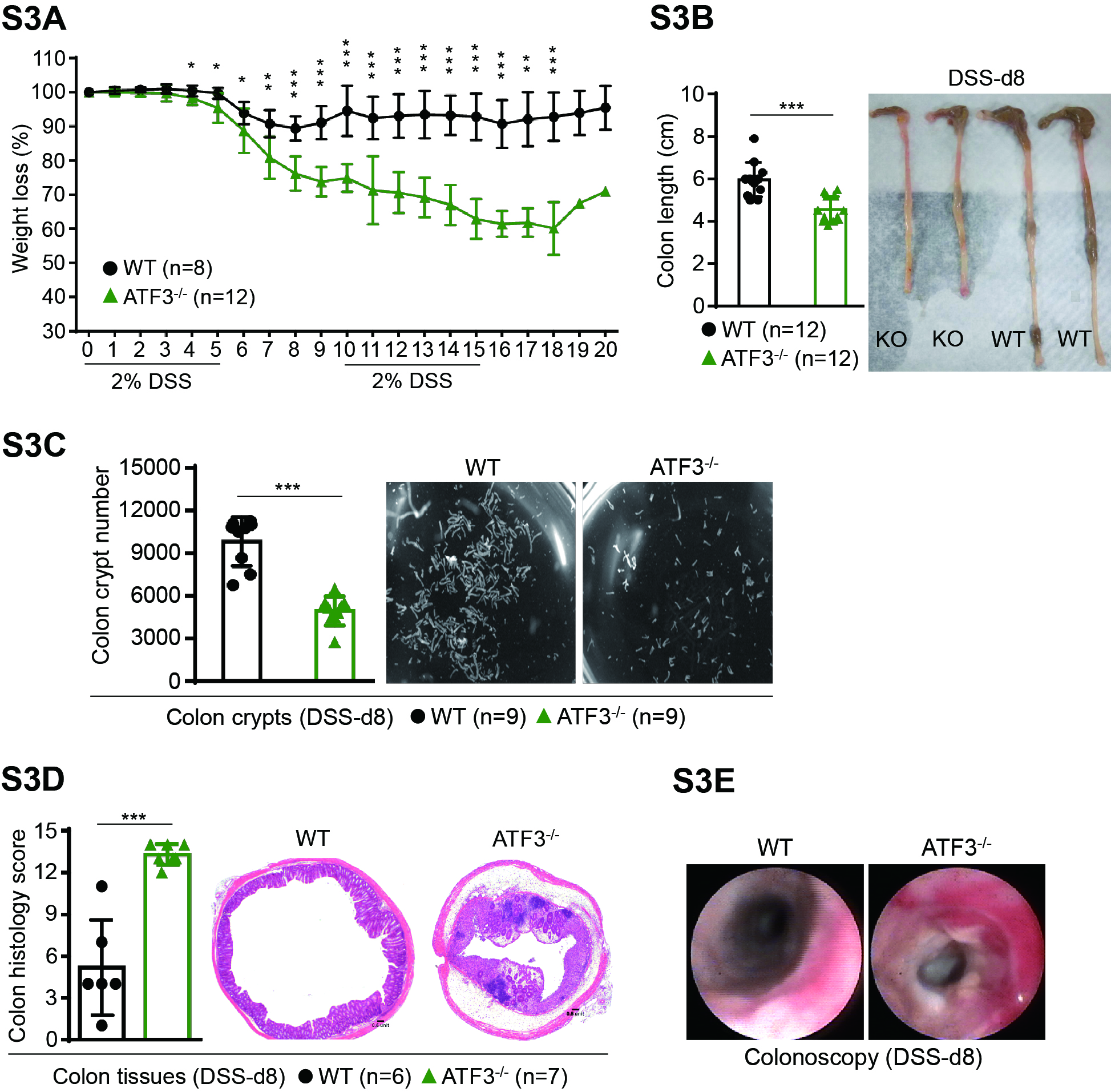

Supplement: Supplementary Figure 3 — ATF3−/− mice were more susceptible to DSS colitis. Analysis of colitis severity during DSS treatment. (A) Percentage of body weight loss during DSS colitis. (B) Colon length, (C) total colon crypt numbers, (D) colon tissue histology scores based on hematoxylin and eosin (H and E) staining, and (E) colonoscopic appearance were analyzed at the indicated day post DSS treatment. Results shown were from two independent experiments and “n” refers to the number of mice used for analysis. Statistical analysis was done using Multiple T-test on Prism software. *P < 0.05, **P < 0.005, ***P < 0.0005. [file Image_3.JPEG]

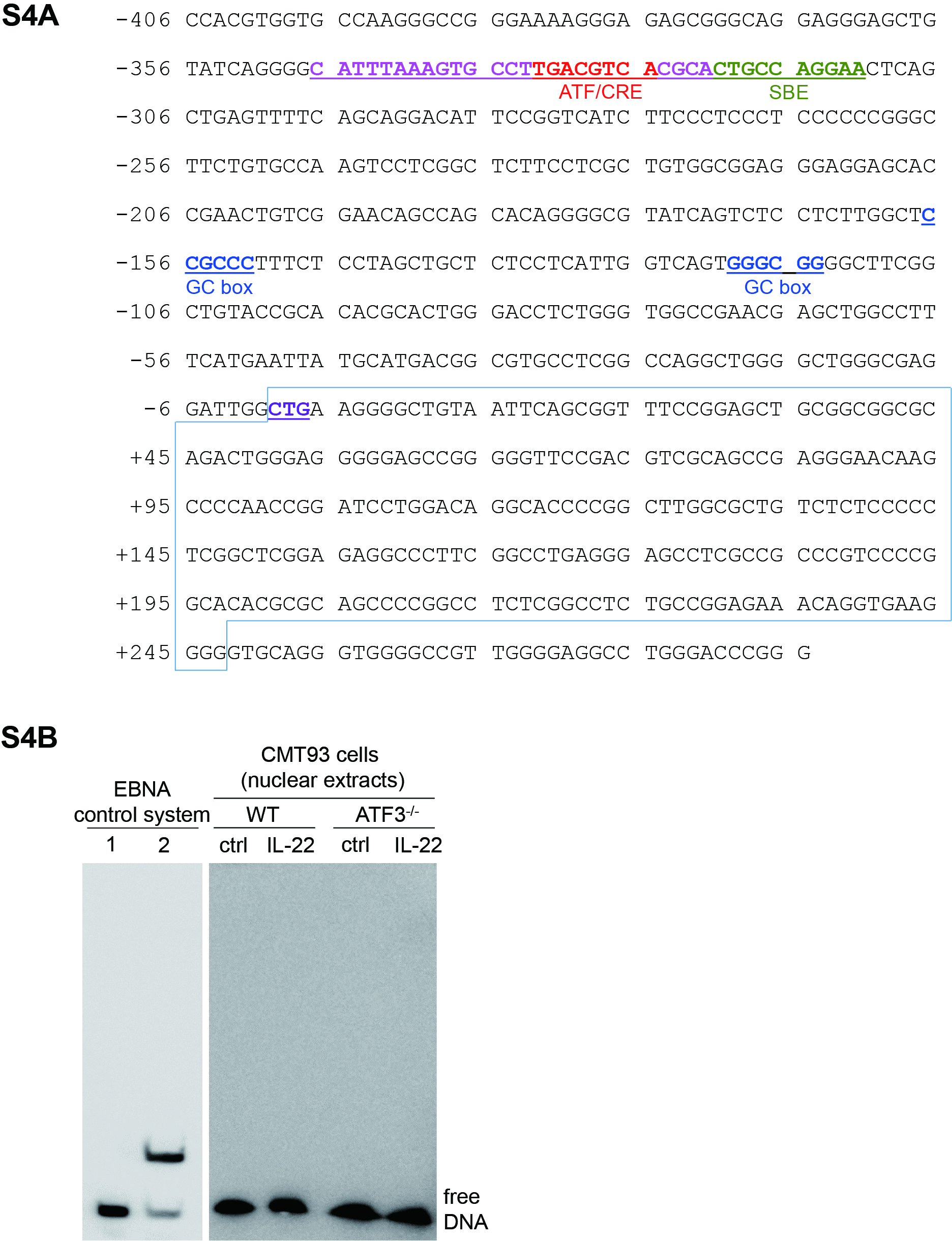

Supplement: Supplementary Figure 4 — ATF3 does not target the STAT3 promoter during IL-22 signaling in CMT93 epithelial cells. (A) Sequence of the mouse STAT3 promoter. Oligonucleotide probe (underlined), containing ATF/CRE binding site (shown in red) and STAT-binding element (SBE, shown in green) in the STAT3 promoter, was used for EMSA experiment. CTG (indicated in purple) is the transcriptional initiation site. GC box (shown in blue) is indicated. (B) EMSA assay, control system: Lane #1, only biotin-labeled 60 bp duplex bearing the EBNA-1 binding sequence showing only free DNA. Lane #2, biotin-labeled 60 bp duplex bearing the EBNA-1 binding sequence and EBNA extract showing DNA-protein complex shift. In assay with CMT93 cells, EMSA was performed with biotinylated STAT3 promoter probe and nuclear extracts prepared from WT or ATF3−/− CMT93 cells with or without IL-22 stimulation (50 ng/ml, 10 min after 5 h of serum starvation). EBNA: Epstein-Barr Nuclear Antigen. Results shown were representative of two independent experiments. [file Image_4.JPEG]

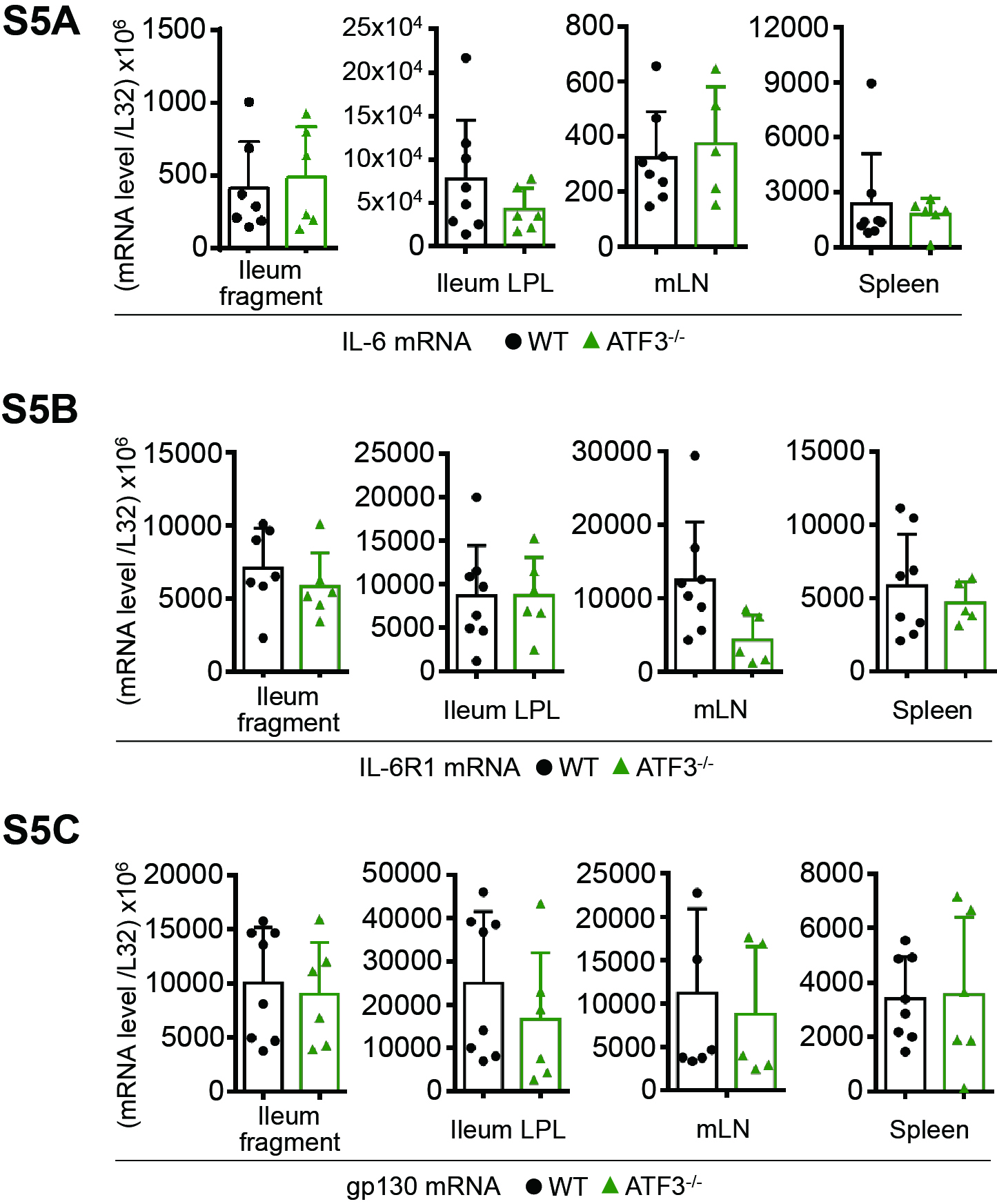

Supplement: Supplementary Figure 5 — ATF3 deficiency in mice does not affect mRNA levels of IL-6, IL-6R1 and gp130 in intestinal compartments. Quantitative real-time PCR analysis of (A) IL-6, (B) IL-6R1, and (C) gp130 mRNA levels in freshly isolated tissues from different intestinal compartments and abdominal organs. Samples of mesenteric lymph nodes (mLN) and spleen were used for comparison. Results shown were combined from two independent experiments and “n” refers to the number of mice used for analysis. No statistical difference between wild-type and ATF3−/− mice was detected. [file Image_5.JPEG]
